# Global overview of multiple sclerosis care units: an international cross-sectional survey

**DOI:** 10.3389/fneur.2026.1812690

**Published:** 2026-06-03

**Authors:** Per Soelberg Sörensen, Paola F. Zaratin, Mario Alberto Battaglia, Gilles Edan, Sara Samadzadeh, Letizia Leocani, Jefferson Becker, Jose Flores, Fernando Hamuy, Edgardo Cristiano, Liliana Patrucco, Xavier Montalbán, Hans-Peter Hartung, Giancarlo Comi, Oscar F. Fernandez

**Affiliations:** 1 Danish Multiple Sclerosis Center, Department of Neurology, Copenhagen University Hospital Rigshospitalet, Copenhagen, Denmark; 2 Fondazione Italiana Sclerosi Multipla, Genoa, Italy; 3 Department of Neurology, Rennes University Hospital, Rennes, France; 4 Charite – Universitätsmedizin Berlin, Corporate Member of Freie Universität Berlin and Humboldt-Universität zu Berlin, Experimental and Clinical Research Center, Berlin, Germany; 5 Faculty of Medicine, Vita-Salute San Raffaele University, Milan, Italy; 6 Experimental Neurophysiology Unit, Institute of Experimental Neurology (INSPE) - IRCCS San Raffaele Scientific Institute, Milan, Italy; 7 Department of Neurology and Psychiatry, Pontifical Catholic University of Rio Grande do Sul, Porto Alegre, Brazil; 8 National Institute of Neurology and Neurosurgery, CAMINEMOS.MX, Mexico City, Mexico; 9 National Multiple Sclerosis Center IMT Hospital, Ministry of Health of Paraguay, Asuncion, Paraguay; 10 Multiple Sclerosis Centre Buenos Aires (CemBA), Buenos Aires, Argentina; 11 Servei de Neurologia and Centre d'Esclerosi Múltiple de Catalunya (Cemcat), Institut de Recerca Vall d'Hebron (VHIR), Hospital Universitari Vall d'Hebron, Universitat Autònoma de Barcelona, Barcelona, Spain; 12 Center for Networked Biomedical Research on Neurodegenerative Diseases (CIBERNED) - ISCIII, Madrid, Spain; 13 Department of Neurology, Heinrich-Heine-University Düsseldorf, Düsseldorf, Germany; 14 Brain and Mind Centre, University of Sydney, Sydney, NSW, Australia; 15 Department of Neurology, Palacky University Olomou, Olomouc, Czechia; 16 Universita Vita Salute San Raffaele, Milan, Italy; 17 Department of Pharmacology, Faculty of Medicine, University of Malaga, Institute of Biomedical Research of Malaga (IBIMA), Malaga, Spain

**Keywords:** multiple sclerosis care units, multidisciplinary care, global overview, questionnaire survey, survey

## Abstract

**Background:**

Multiple sclerosis care units (MSCUs) are a reference model for multidisciplinary MS care, yet comparable global data on their structure, resources, and performance remain limited.

**Objective:**

To characterize the global landscape of MS centers and evaluate compliance with multidisciplinary standards using a simplified, scalable classification framework.

**Methods:**

An international cross-sectional survey (2020–2021) collected validated responses from 198 MS centres across 38 countries. Comparative analyses focused on 168 hospital-based centres. MSCUs were initially classified as type I or II based on fulfilment of ≥80% or 70–79% of 22 predefined structural and functional criteria. To enhance reproducibility, a complementary model was developed using 16 essential criteria, one point per fulfilled item; centres meeting ≥14 criteria qualified for classification. Additional thresholds for annual MS patient volume and neurologist staffing differentiated MSCU-I from MSCU-II. Analyses were stratified by region and institution type.

**Results:**

Academic centers reported higher workloads: mean annual treatments 1,359.6 vs. 779.3 (*p* = 0.0003) and treated relapses 181.4 vs. 84.1 (*p* < 0.0001). Operational data systems were widely used (administrative 81.5%, registries 85.7%), with stronger regional than institutional gradients: Western Europe had greater adoption (e.g., cost-accountability 67.0% vs. 20.6% in Latin America). Serious adverse events averaged 28.9/year with no regional or institutional differences. Diagnostic and therapeutic capacities (EPs, OCT, high-efficacy DMTs, ASCT) varied markedly by region. Among the 168 hospital centers, 141 met inclusion criteria; 120 (71.4%) met MSCU-I and 21 (12.5%) met MSCU-II criteria, while 27 (16.1%) did not meet the MSCU standards. Region was a stronger predictor of MSCU-I status than academic affiliation (*p* = 0.0001 and *p* = 0.0458, respectively). Higher national GDP and health expenditure correlated with MSCU-I designation. A minority of mid-volume units (≈500–999 patients/year) with essential staffing may warrant a pragmatic MSCU-III category for benchmarking.

**Conclusion:**

We provide the broadest overview to date of MSCU structure and operations and propose a practical, reproducible 16-criterion classification framework. Regional context outweighs academic status in predicting MSCU adequacy. Because the dataset is driven predominantly by Europe and Latin America, extrapolation of these findings to under-represented regions, particularly North America and the Rest of the World, should be made with caution. The framework supports practical benchmarking, certification, and targeted capacity-building to reduce regional disparities in diagnostics, information systems, and access to high-efficacy therapies.

## Highlights

- This is the first global cross-sectional survey characterizing the structure and operations of multiple sclerosis care units (MSCUs).
- A total of 198 validated MS Centers from 38 countries participated, with comparative analyses focused on 168 hospital-based centers.
- Substantial variability was observed in patient volume, staffing, infrastructure, and academic activity across regions and institution types.
- MSCUs were initially classified as type I or II based on fulfillment of ≥80% or 70–79% of 22 predefined criteria.
- A stricter complementary classification system using 16 essential criteria and quantitative thresholds was developed for reproducible benchmarking.
- The dual classification approach supports both consensus-based and standardized evaluations of MSCU quality across heterogeneous settings.

## 1 Introduction

Multiple sclerosis (MS) is a chronic, immune-mediated condition that affects the central nervous system (CNS), with heterogeneous symptoms, clinical manifestations and disease progression. The disease affects more than 2.8 million people globally and poses significant challenges for healthcare systems. These challenges are not only due to the disease’s complex biology and the long-term disabilities it may cause, but also because of the need for lifelong, coordinated, and multidisciplinary care to achieve optimal outcomes for patients (1, 2).

Considering these demands, the concept of the multiple sclerosis care unit (MSCU) has gained growing attention as a promising care model (3). MSCUs are specialized organizational models designed to integrate various healthcare professionals and resources within a structured framework for MS management (3). Their core objectives include promoting timely and accurate diagnosis, offering individualized treatment strategies, ensuring continuous follow-up, and delivering person-focused care in line with evolving standard of care (4). While international guidelines increasingly emphasize multidisciplinary approaches in MS management, standardized data on how MSCUs are organized and operate across different countries and healthcare system settings are still limited (4, 5).

Previous surveys from individual countries or regions have shed light on access to MS-related services (6), but no study to date has systematically explored the structure, resources, and care delivery roles of MSCUs on a global scale using consistent methodology. In addition, the way in which institutional factors (i.e., academic status) or broader contextual elements (i.e., local healthcare infrastructure) impact the capacity of MSCUs to provide integrated, high-quality care is not well understood (3).

To address this gap, we conducted a global cross-sectional survey of MS specialists across five key regions: Western Europe, Other European Countries, North America, Latin America, and the Rest of the World. Using a validated, multidimensional questionnaire, we examined how MS-centers are structured, staffed, and equipped; what diagnostic and treatment capacities they offer; how they manage information and patient-centered services; and to what extent they follow established multidisciplinary care standards (MSCU-I and MSCU-II).

This study provides the most detailed global overview to date of how MS-centers are organized in different healthcare environments. By presenting comparative benchmarks on infrastructure, clinical processes and care models, and by identifying key regional disparities, our findings aim to support clinicians, healthcare authorities, and policy makers in advancing the quality and accessibility of MS care worldwide.

## 2 Materials and methods

### 2.1 Study design

The present multicenter, international, cross-sectional descriptive study was initiated by the European Charcot Foundation (ECF) and conducted in collaboration with the Italian Multiple Sclerosis Society Foundation (FISM), and the MULTI-ACT project. The primary objective was to characterize the organizational structure, clinical practices, and resource allocation of MS-centers across diverse healthcare settings worldwide. The study design aimed to provide a representative overview of MS-centers operations through a standardized global survey. Since the study did not involve personal or animal data, ethical approval was obtained solely from local ethics committees or institutional bodies, as required by each participating center.

### 2.2 Questionnaire development and validation

The MSCU survey instrument was based on a validated tool initially developed for the Spanish national network of Reference Centers, Services and Units (CSUR) under the Spanish Ministry of Health (7–9). The questionnaire was further adapted and refined through consultation with an international panel of MS specialists, drawing from existing international guidelines and expert consensus. The final instrument underwent multiple iterations to ensure cross-cultural relevance, clarity, and comprehensiveness. Content validity was established through consensus among experts from multiple countries.

The final questionnaire covered 11 thematic domains that represent the key components of multidisciplinary MS care (Supplementary material, Annex I):

1 General Description of Participating Organizations
2 Geographic and Organizational Distribution
3 Patient Volume, Staffing, and Academic Activity
4 Clinical, Diagnostic, and Therapeutic Resources
5 Multidisciplinary Resources and Infrastructure
6 Operational Characteristics of MSCUs
7 Access to External Supporting Resources
8 Communication Systems
9 Compliance with MSCU Criteria
10 Implementation of Patient Satisfaction and Patient-Reported Outcomes (PROs)
11 Economic Context and MSCU Classification

### 2.3 Definition of essential criteria for MSCU classification

To establish an objective basis for classifying MS Centers, we predefined two designation levels: MSCU-I, referring to a complete multidisciplinary unit; and MSCU-II, corresponding to a unit meeting the minimum multidisciplinary requirements (Supplementary material, Annex II).

To identify the essential criteria for classification, we conducted a criterion reduction analysis using a previously developed 22-item framework. Each criterion was systematically removed in isolation, and the resulting impact on MSCU-I and MSCU-II classification outcomes was assessed. Criteria whose exclusion modified the classification results were considered essential.

Sixteen out of the 22 original criteria were identified as essential for at least one classification level, indicating their critical role in defining care complexity. These included MS patient volume, number of neurologists, number of nurses, presence of physiotherapists, access to MRI, immunology services, neuropsychology, rehabilitation services, and clinical psychology, as well as involvement in research, continuing medical education (CME) (for MSCU-I), availability of a day hospital, outpatient consultation facilities, Extended Disability Status Scale (EDSS) documentation, access to disease-modifying therapies (DMTs), patient registries, and patient satisfaction assessment. Additionally, the number of nurses and communication systems were essential for at least one classification level.

Six criteria were found to be non-essential, as their exclusion did not impact on the classification outcomes for either MSCU-I or MSCU-II: fluid manipulation area, biobanking facilities, administrative data systems, cost accountability systems and, depending on the classification level, number of nurses, CME, and communication systems.

Based on the refined list of essential indicators, we developed an automated scoring algorithm to enable standardized classification. The scoring system includes 16 essential criteria, each contributing 1 point if fulfilled. A score of ≥14 was required for classification as either MSCU-I or MSCU-II. The threshold of ≥14 was chosen to maintain robustness and scalability across different health systems. In addition, MSCU-I designation required fulfillment of minimum thresholds for MS patient volume (≥1,500 annually), number of neurologists (≥5), and number of nurses (≥4). For MSCU-II classification, the thresholds were ≥1,000 patients and ≥3 neurologists, with mandatory availability of a physiotherapist and MRI access.

### 2.4 Participants and sampling strategy

Participants included neurologists and MS-centers known to provide specialized care for individuals with MS. Eligible organizations were identified through the ECF network, regional MS societies, professional neurological associations, and expert referrals. Invitation emails outlined the study objectives, voluntary participation terms, data confidentiality, and ethical safeguards.

### 2.5 Data collection

The survey was distributed electronically to approximately 500 MS-centers globally between 2020 and 2021. Responses were collected via a secure online platform. A 90-day response period was provided, with follow-up reminders and technical support issued to improve participation. Due to technical issues in early distribution phases, the platform was modified, and the survey was redistributed up to four times with extended deadlines.

Regional participation varied. Some North American (USA and Canada) centers declined due to data protection regulations, and several countries classified under “Rest of the World” contributed limited responses.

The Geographical Classification of Countries was grouped into five macro-regions.

• *Western Europe:* Austria, Belgium, Denmark, Finland, France, Germany, Greece, Italy, Norway, Portugal, Spain, Sweden, Switzerland, United Kingdom.

• *Other European Countries:* Bulgaria, Czech Republic, Estonia, Hungary, Lithuania, Poland, Romania, Russia, Serbia, Slovenia, Turkey.

• *North America:* United States, Canada.

• *Latin America:* Argentina, Brazil, Chile, Colombia, Guatemala, Honduras, Mexico, Paraguay, Peru, Uruguay.

• *Rest of the World:* Australia, Israel, Japan, Lebanon, Tunisia.

### 2.6 Data quality assurance

Data integrity was maintained through a two-step quality control process. Initial removal of duplicates and inconsistent entries yielded 215 valid records, followed by further refinement to produce a dataset of 204 responses. Final inclusion criteria excluded non-clinical centers and entries lacking essential variables (e.g., institution type, country, patient volume), resulting in a final analytical sample of 198 organizations from 38 countries. Data completeness and consistency were systematically reviewed. Clarifications and data corrections were pursued through follow-up communication with respondents when necessary. Missing data were treated using a complete case approach. Variables with a high proportion of missing values were excluded from specific comparative analyses.

### 2.7 Statistical analysis

Descriptive statistics were used to summarize variables. Categorical data were reported as frequencies and percentages; continuous variables were expressed as the mean, median, standard deviation (SD), or interquartile range (IQR), depending on the data distribution. Comparisons between academic and non-academic institutions and across regions were performed using chi-square (*χ*^2^), Fisher’s exact, Mann–Whitney *U*, or Kruskal–Wallis tests, with *p* < 0.05 being considered statistically significant. Multivariate logistic and linear regression models were used to explore associations between MSCU characteristics and key outcomes, adjusting for confounding variables such as region, institution type, and patient volume.

### 2.8 Software for data analysis and visualization

Data curation was conducted in Microsoft Excel (Microsoft Corp., Redmond, WA, USA). Statistical analyses and visualizations were performed using Python (v3.10; pandas, matplotlib, seaborn), R (v4.2.2), and the SPSS statistical package (v28.0; IBM Corp., Armonk, NY, USA). Custom scripts were used to generate heatmaps, stacked bar plots, and scatter plots.

## 3 Results

### 3.1 General description of participating organizations

**Figure 1:**
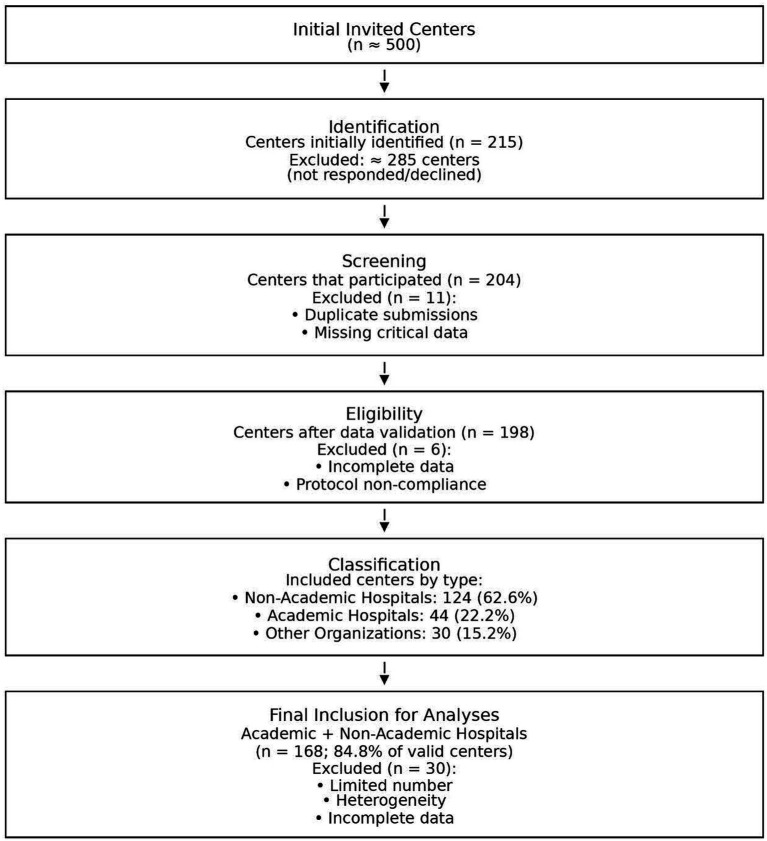
Flow chart of center selection and inclusion process.

A total of 204 MS care organizations from 42 countries participated in the survey, representing an estimated 40% response rate from the ~500 centers initially invited. After a rigorous quality control process, 198 organizations from 38 countries were included in the final analysis. Among these, 124 (62.6%) were classified as non-academic hospitals, 44 (22.2%) as academic hospitals, and 30 (15.2%) as other types of organizations. Due to their limited number, heterogeneity, and incomplete data, this latter group was excluded from most stratified analyses (Figure 1). Consequently, comparative analyses were primarily conducted on the 168 organizations identified as academic or non-academic hospitals, together accounting for 84.8% of the final dataset (Table 1; Supplementary Table S1).

**Table 1:** Distribution of MS-centers by region and type of organization.

Region	Academic hospital *n* (%)	Non-academic hospital *n* (%)	Other *n* (%)	Total *n* (%)
Western Europe	23 (22.1)	75 (72.1)	6 (5.8)	104 (52.5)
Other European Countries	13 (38.2)	15 (44.1)	6 (17.6)	34 (17.2)
North America^a^	1 (20.0)	4 (80.0)	0 (0.0)	5 (2.5)
Latin America	5 (10.2)	27 (55.1)	17 (34.7)	49 (24.7)
Rest of the World	2 (33.3)	3 (50.0)	1 (16.7)	6 (3.0)
TOTAL	44 (22.2)	124 (62.6)	30 (15.2)	198 (100.0)

a The only academic hospital in North America did not provide complete staff data; therefore, its variables are shown as N.A. in Table 2.

### 3.2 Geographic and organizational distribution

**Figure 2:**
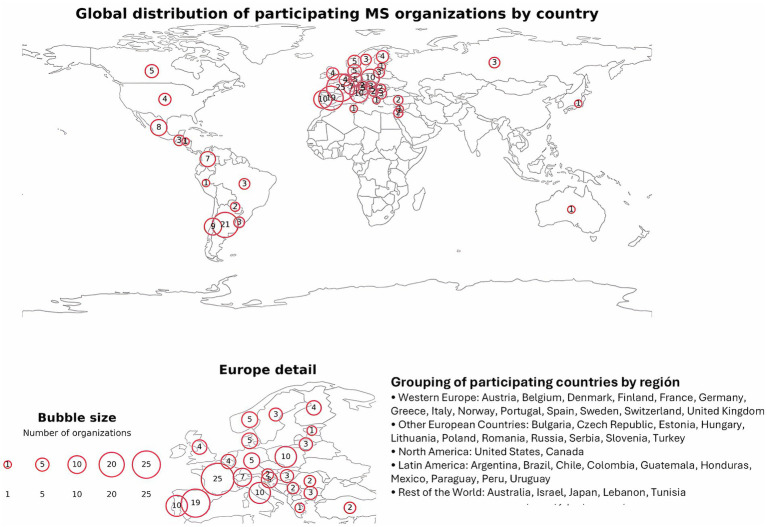
Map of MS centers participation (*n* = 198) by country (38).

Of the 198 validated MS-Centers, distribution was across five predefined global regions: Western Europe, Other European Countries, North America, Latin America, and Rest of the World. Western Europe represented the largest share, with 104 organizations (52.5%), followed by Latin America (*n* = 49, 24.7%), Other European Countries (*n* = 34, 17.2%), Rest of the World (*n* = 6, 3.0%), and North America (*n* = 5, 2.5%) (Table 1, Supplementary Table S1). Within Western Europe, France (*n* = 25), Spain (*n* = 19) and Italy (*n* = 10) had the most centers; in Latin America, Argentina accounted for the highest number (*n* = 21). Figure 2 provides an overview of the geographic distribution and regional classification of the MS Centers.

The distribution of academic and non-academic hospitals varied by region. Non-academic hospitals predominated in Western Europe (72.1%) and Latin America (55.1%). Although academic centers were more common in Other European Countries and Rest of the World than in other regions, they still comprised a minority (38.2 and 33.3%, respectively). In North America, most centers were non-academic (80.0%) (Table 1). Supplementary Figure S1 illustrates the proportional distribution of academic and non-academic hospitals by region.

### 3.3 Patient volume, staffing, and academic activities

#### 3.3.1 Annual patient volume per center

Annual MS patient volume varied substantially by region and institutional type. Academic hospitals in Western Europe reported the highest mean ± standard deviation (SD) volumes (2,228 ± 901 patients/year), followed by Other European Countries (1,875 ± 704 patients/year) and Latin America (1,133 ± 854 patients/year) (Supplementary Table S2). Non-academic hospitals also managed considerable patient mean ± SD numbers, ranging from 1,426 ± 853 patients/year in Western Europe to 860 ± 745 patients/year in Latin America. Limited data from North America and the Rest of the World indicated moderate to low mean volumes (North America: 2,000 ± 894 patients/year for academic and 918 ± 329 patients/year for non-academic institutions). These distribution patterns suggest that Western Europe maintains the highest throughput, especially within academic settings, while Latin America and the Rest of the World report lower volumes.

#### 3.3.2 Staffing and human resources

Staffing levels varied considerably by region and type of hospital (Table 2). On average, academic hospitals reported a higher number of staff members across most professional roles. In Western Europe, academic MS-Centers had a mean ± SD of 6.5 ± 2.69 neurologists, 4.25 ± 3.24 nurses, and 2.7 ± 1.92 physiotherapists, versus 5.86 ± 4.95, 5.73 ± 12.85, and 2.5 ± 2.99, respectively, in non-academic facilities. In Other European Countries, academic centers staffed more neurologists (6.92 ± 5.2) than non-academic centers (3.43 ± 1.87). In Latin America, staffing levels were more variable, though academic institutions still had a greater mean number of neurologists (7.0 ± 3.83 versus 4.53 ± 3.48).

**Table 2:** Distribution of MS-center staff by region and type of hospital (*n* = 168).

Region	Neurologists (mean ± SD)		Nurses (mean ± SD)		Physiotherapists (mean ± SD)	
	Academic hospital (*n* = 44)	Non-academic hospital (*n* = 124)	Academic hospital (*n* = 44)	Non-academic hospital (*n* = 124)	Academic hospital (*n* = 44)	Non-academic hospital (*n* = 124)
Western Europe	6.5 ± 2.69	5.86 ± 4.95	4.25 ± 3.24	5.73 ± 12.85	2.7 ± 1.92	2.5 ± 2.99
Other European Countries	6.92 ± 5.2	3.43 ± 1.87	4.17 ± 2.29	3.21 ± 1.93	2.42 ± 1.38	2.07 ± 0.83
North America^a^	N. A.	5.5 ± 3.87	N. A.	3.58 ± 1.15	N. A.	0.62 ± 0.48
Latin America	7.0 ± 3.83	4.53 ± 3.48	2.75 ± 2.22	4.6 ± 9.52	3.5 ± 1.91	5.0 ± 7.69
Rest of the World	6.0 ± N. A.	2.5 ± 0.71	5.0 ± N. A.	3.0 ± 2.83	5.0 ± N. A.	1.5 ± 0.71

N.A., not available; SD, standard deviation.

a The only academic hospital in North America did not provide complete staff data.

Two-way ANOVA indicated no significant effect of region (*p* = 0.77) and no significant region per institution interaction (*p* = 0.38). Nevertheless, there was a non-significant trend towards higher staffing in academic hospitals (*p* = 0.097), consistent with the observed descriptive data. Supplementary Figure S2 presents a heatmap summarizing staffing patterns by region and institution. The multidisciplinary approach to MS care is illustrated by access to other professionals including neuropsychologists (*n* = 156), clinical psychologists (*n* = 123), rehabilitation physicians (*n* = 133), speech therapists (*n* = 118), and occupational therapists (*n* = 99).

#### 3.3.3 Professional and academic activities

**Figure 3:**
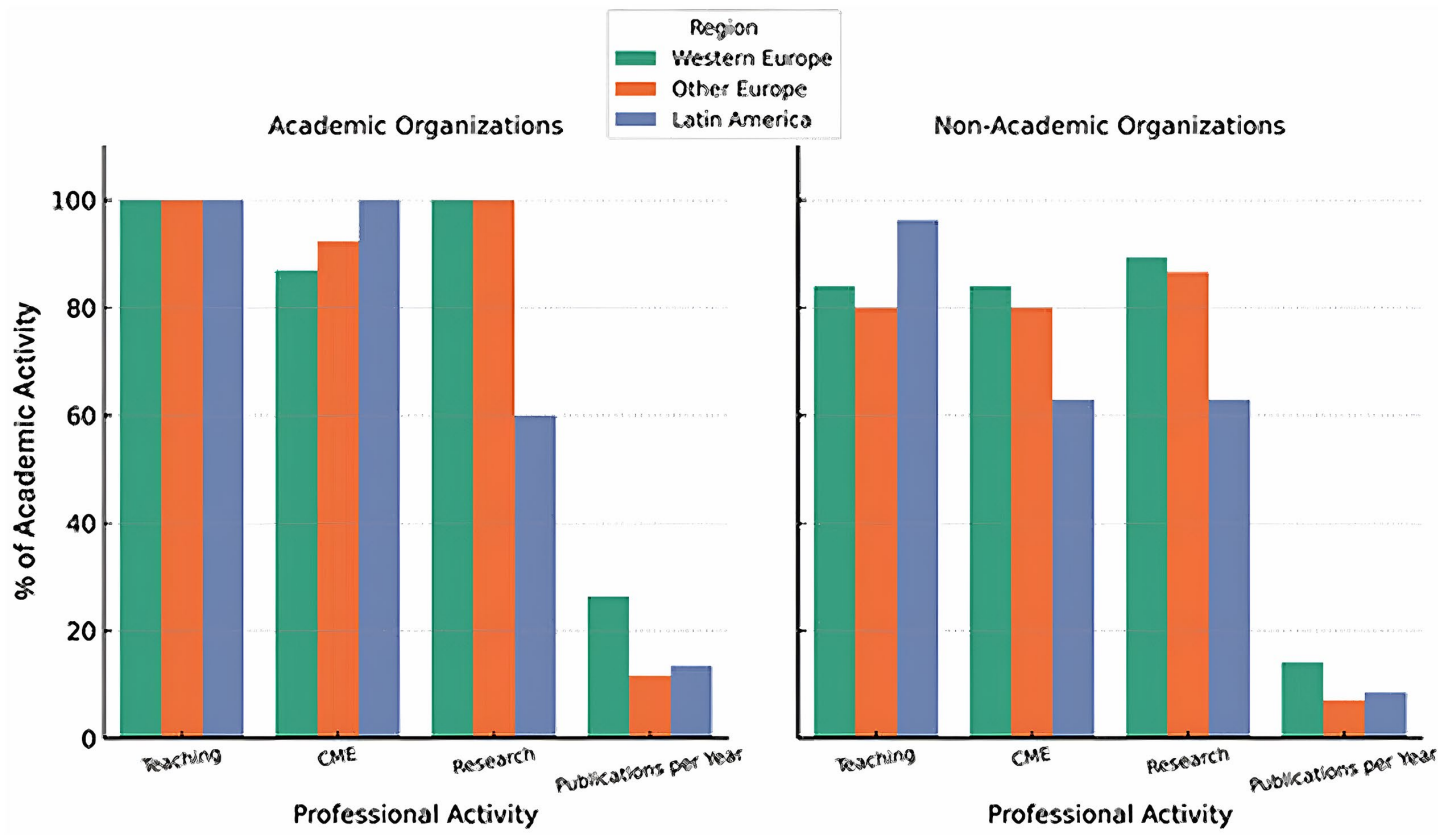
Correlation between professional activities (teaching, CME, research, publications) by hospital type and region (*n* = 168). Participation in academic activities in MS-Centers by hospital type and region. The figure shows the percentage of centers engaged in teaching, continuing medical education (CME), and research (binary variables), along with the mean number of annual publications (continuous variable). Comparisons are presented by institution type (academic vs. non-academic) and region (Western Europe, Other Europe, and Latin America).

Involvement in academic activities, including teaching, continuing medical education (CME), research, and publication, was consistently greater among academic institutions and exhibited regional differences (Supplementary Table S3, Figure 3). In Western Europe, more than 85% of MS-Centers participated in teaching, CME, and research. Academic centers in this region reported a mean of 26.4 publications annually, compared to 14.1 in the case of non-academic centers (*p* = 0.0007). In Other European Countries, mean publication numbers were lower (11.7 versus 7.1), but participation in teaching and CME remained high. In Latin America, while teaching and CME were also common, engagement in research (53.1%) and publication output in terms of mean publication numbers were lower (academic: 13.5 versus non-academic: 8.7).

Both regional location and institution type significantly influenced the number of scientific publications as determined by two-way ANOVA (*p* < 0.05). There was no significant interaction between these factors, indicating that each independently affected publication output. Research activity also varied significantly across regions (*χ*^2^ = 18.91, *p* = 0.0007), with much higher engagement observed in European regions compared to Latin America. In contrast, teaching and CME participation remained consistently high, without significant variation by region or institutional category.

### 3.4 Clinical, diagnostic and therapeutic resources

#### 3.4.1 Clinical resources

Among the 164 MS-Centers out of 198 participating centers (82.8%) that provided data on clinical assessment tools, the Expanded Disability Status Scale (EDSS) was the most frequently employed (78.3%). In 30.3% of the centers, EDSS was used in combination with the Multiple Sclerosis Functional Composite (MSFC). Only 5.3% of the organizations reported incorporating broader tools such as the Symbol Digit Modalities Test (SDMT), Timed 25-Foot Walk (T25FW), Nine-Hole Peg Test (9HPT), or Brief International Cognitive Assessment for MS (BICAMS), suggesting a limited adoption of comprehensive evaluation strategies. Notably, 17.2% of the centers did not specify the tools employed for clinical assessment.

#### 3.4.2 Diagnostic resources

Access to diagnostic tools differed by region and hospital type (*n* = 168) (Supplementary Table S4, Supplementary Figure S3). Magnetic Resonance Imaging (MRI) and blood testing were available in over 90% of units in Western Europe and selected regions, though their presence was not consistently uniform worldwide. Greater variability was observed for cerebrospinal fluid (CSF) studies, evoked potentials (EPs), and optical coherence tomography (OCT). Western Europe had the most extensive diagnostic resources, with high rates of access across all modalities, especially in academic centers. Conversely, North America and Latin America showed more heterogeneous patterns of availability. Notably, OCT was used in only 25% of non-academic institutions in North America. Fisher’s exact tests revealed significant institutional differences regarding blood test access in Western Europe (*p* = 0.012) and EPs in Other European Countries (*p* = 0.003). These findings highlight ongoing differences in diagnostic access.

#### 3.4.3 Therapeutic resources

The use of disease-modifying therapies (DMTs) was widespread across all regions and hospital types (*n* = 168), although usage patterns showed greater variation by region than by hospital category (Table 3, Supplementary Tables S5–S11, Supplementary Figures S4–S6). DMTs were grouped by efficacy as follows:

- *Low–moderate efficacy:* interferons, glatiramer acetate, teriflunomide
- *Moderate efficacy:* fumarates, sphingosine 1-phophate (S1P) receptor modulators
- *High efficacy:* monoclonal antibodies (e.g., natalizumab, ocrelizumab, alemtuzumab, ofatumumab), cladribine, mitoxantrone
- *Autologous hematopoietic stem cell transplantation* (*ASCT*) was assessed separately

**Table 3:** MS therapy use by efficacy level, region and hospital type (*n* = 168).

Region	Low-moderate efficacy (%)		Moderate efficacy (%)		High efficacy (%)		ASCT (%)	
	Academic hospital (*n* = 44)	Non-academic hospital (*n* = 124)	Academic hospital (*n* = 44)	Non-academic hospital (*n* = 124)	Academic hospital (*n* = 44)	Non-academic hospital (*n* = 124)	Academic hospital (*n* = 44)	Non-academic hospital (*n* = 124)
Western Europe	90.0	88.0	75.0	73.0	116.5^a^	119.3^a^	56.5	61.3
Other European Countries	85.0	83.0	70.0	68.0	93.5	93.0	38.5	40.0
North America	88.0	86.0	78.0	76.0	65.0	138.0^a^	0.0	75.0
Latin America	80.0	78.0	68.0	66.0	90.0	59.1	40.0	11.1
Rest of the World	75.0	73.0	60.0	58.0	95.0	43.0	50.0	0.0

ASCT, autologous hematopoietic stem cell transplant.

a Percentages exceeding 100% reflect the fact that individual MS-Centers often use more than one high-efficacy therapy. As the analysis counted each distinct treatment reported per center, the cumulative percentage may surpass 100% in regions with broader access to multiple agents.

No significant differences were observed in the use of specific therapies between academic and non-academic centers (*p* > 0.14 in all cases; Supplementary Table S5). However, multinomial logistic regression showed that geographic region, rather than institution type, was the primary determinant for access to moderate- and high-efficacy DMTs (*p* < 0.01).

Western Europe and North America exhibited the highest rates of high-efficacy DMT use, with cumulative percentages exceeding 115%, reflecting widespread use of multiple high-efficacy agents within individual centers. In contrast, centers located in Latin America and the Rest of the World were significantly less likely to use high-efficacy treatments (adjusted OR = 0.46; 95% CI [0.25–0.84]; *p* = 0.012), highlighting persistent regional treatment disparities.

Access to and use of ASCT varied substantially by region. ASCT was most often reported by non-academic hospitals in North America (75%) and by academic centers in Latin America (40%). In Western Europe, reported use was lower, with availability in 56.5% of academic centers and 61.3% of non-academic hospitals. In Other European Countries, rates were further reduced (38.5 and 40.0%, respectively), and in the Rest of the World, ASCT was rarely available. These results show that access to advanced therapies such as ASCT depends more on regional healthcare resources and infrastructure than on academic status alone.

### 3.5 Multidisciplinary resources and infrastructure

**Figure 4:**
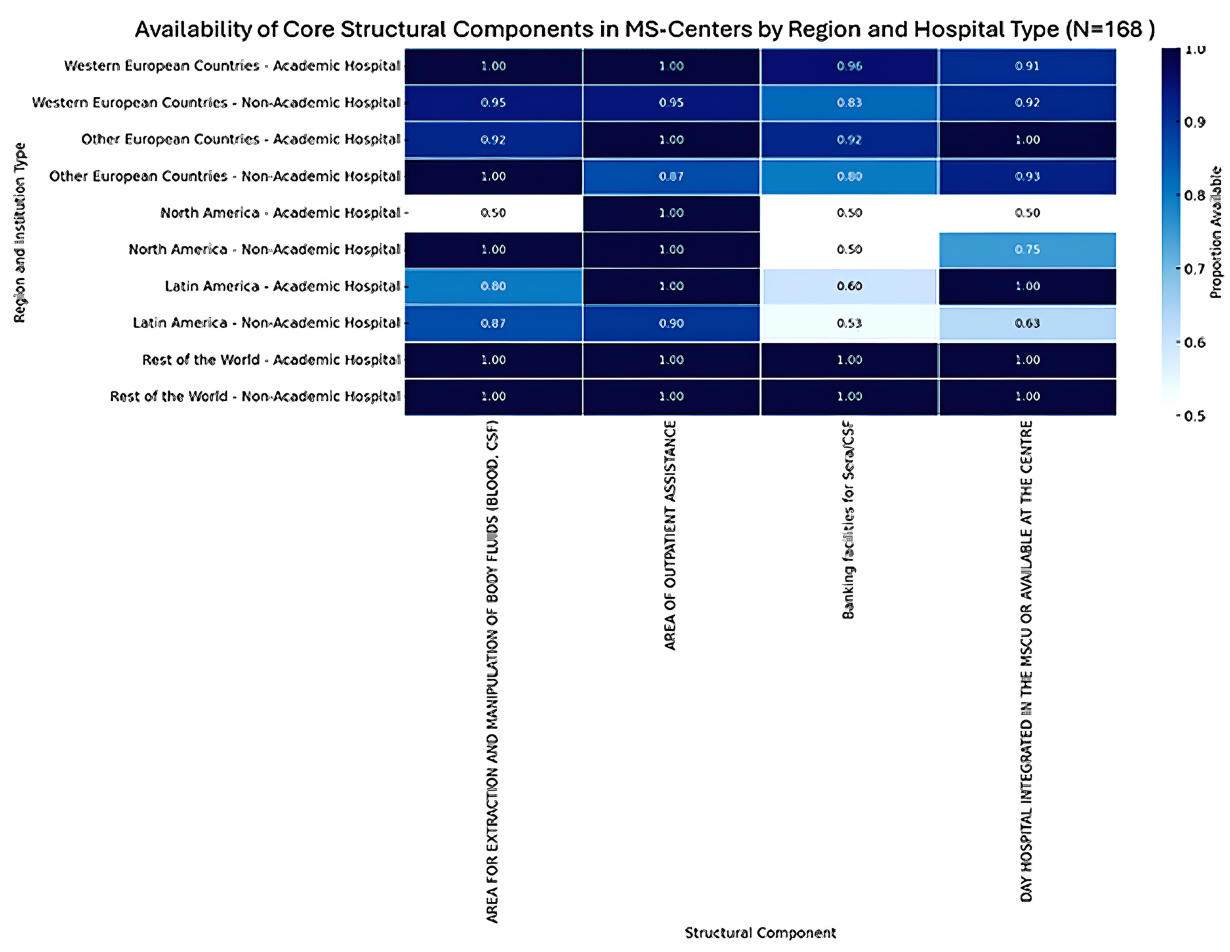
Availability of core structural components in MS-centers by region and hospital type (*n* = 168). Heatmap illustrating the availability of core structural components across multiple sclerosis centers, stratified by region and type (academic vs. non-academic hospitals). Values represent the proportion of units in each group reporting availability of: (1) Day hospital integration; (2) Biological fluid extraction area; (3) Outpatient assistance area; and (4) Banking facilities for sera/CSF. Darker shades reflect higher availability.

Availability of key structural elements within MS-Centers differed markedly by geographic region and hospital type (*n* = 168) (Supplementary Table S12, Figure 4). Academic hospitals generally reported broader access to infrastructure, especially in Western Europe and North America. In these areas, the majority of academic MS-Centers possessed well-developed resources, including day hospital services, dedicated outpatient areas, biological fluid extraction units, and biobanking facilities for serum and CSF.

Academic centers in Western Europe exhibited the most complete infrastructure, with virtually all reporting full access to these core components. While academic institutions in Other European Countries and Latin America were also generally better equipped than non-academic sites, the contrast was less pronounced. By comparison, facilities in the Rest of the World had much more limited infrastructure, and non-academic hospitals in these regions often lacked both outpatient areas and banking facilities for biological samples.

The heatmap in Figure 4 illustrates these differences, highlighting that MS-Centers in Western Europe maintained the strongest structural capacities, irrespective of institution category, while other regions showed more unequal resource allocation. Overall, these results emphasize that regional factors have greater influence than institutional type in shaping the infrastructure available for comprehensive MS care.

### 3.6 Operational characteristics of MS-centers

A total of 12 operational variables were evaluated to describe the functional activities of the 168 MS-Centers (Supplementary Table S13). These variables provide insight into clinical workload, data management strategies, and procedural practices.

The annual number of treatments delivered in outpatient or day-hospital settings (oral or intravenous) averaged 941.7 (SD 899.2; range 100–2,900). Academic centers reported higher means than non-academic centers (1,359.6 versus 779.3; *p* = 0.0003). By region, Western Europe showed the highest activity (1,297.9), and Latin America the lowest (182.4; *p* < 0.0001).

For relapse management, academic centers had more treated exacerbations than non-academic centers (181.4 versus 84.1; p < 0.0001). Regionally, Other European Countries (168.3) and North America (166.7) showed the highest means, while Western Europe was intermediate (114.9), and Latin America the lowest (42.6; *p* < 0.0001). The global mean (SD; range) for serious adverse events was 28.9 (SD 16.4; 25–175), without significant differences by institution (*p* = 0.5068) or region (*p* = 0.2005).

Administrative data were used for internal monitoring in 81.5% of the MS-Centers, with no institutional differences (academic 85.1% versus non-academic 80.2%; n.s.). Western Europe had higher adoption (91.5%) compared with Latin America (67.6%) and Other European Countries (76.7%; *p* = 0.0015).

Cost-accountability systems existed in 50.6% of the MS-Centers and were more common in academic than in non-academic centers (66.0% versus 47.9%; *p* = 0.0538, n.s.). Western Europe showed the highest use (67.0%), and Latin America the lowest (20.6%; p < 0.0001).

Multiple sclerosis patient registries were reported by 85.7% of the centers, with similar proportions in academic and non-academic institutions (85.1% versus 86.0%; n.s.). Regional differences were present (*p* = 0.0185), with Western Europe showing higher coverage (92.6%) than Other European Countries (73.3%) and Latin America (82.4%).

For the Minimal Data Set (MDS) elements, reporting rates were high: patient identification (84.5%), sex (84.5%), date of birth (83.9%), date of diagnosis (82.7%), date of exacerbations (82.7%), and geographic information (72.6%). Diagnostic procedures and coded secondary diagnoses (ICD-9) were recorded by 65.5 and 59.5%, respectively. No institutional differences emerged (n.s. in all cases), whereas some regional disparities were observed for patient identification (*p* = 0.0303) and date of birth (*p* = 0.0157); other items were nonsignificant.

Hospitalization data were variably recorded: admission date (67.9%), discharge date (56.0%), and type of hospitalization (55.4%). Urgent and programmed hospitalizations were documented by 42.9 and 38.1% of the MS-Centers, respectively. Institutional differences were nonsignificant; regionally there were modest differences for discharge date (*p* = 0.0367) and hospitalization type (*p* = 0.0091), while urgent/programmed hospitalization was nonsignificant.

For discharge outcomes, the most documented items included discharge home (54.2%), principal diagnosis (50.0%), death (43.5%), and inter-hospital transfer (38.7%). Institutional differences were nonsignificant. Regionally, death recording was more frequent in Western Europe (54.3%) than in Latin America (38.2%) or Other European Countries (30.0%; *p* = 0.0053). Inter-hospital transfers were also higher in Western Europe (47.9%) compared with Latin America (32.4%) and Other European Countries (26.7%; *p* = 0.0498).

Routine recording of visit dates was reported by 75.0% of the MS-Centers, with no institutional differences (n.s.) but with a regional effect (*p* = 0.0236, higher in Western Europe). The nature of the visit was often documented: scheduled (53.0%) and urgent (50.6%), with no significant differences by institution or region (n.s. in all cases). The most frequent reasons for visit were relapse management (73.2%), DMT initiation/monitoring (71.4%; regional *p* = 0.0062), and diagnostic assessment (68.5%; n.s.).

Finally, the adoption of adverse event classification systems was heterogeneous: ICD-9 (35.7%), WHO Toxicity Grading Scale (24.4%), and MedDRA (15.5%). Institutional differences were nonsignificant; regional contrasts were significant for ICD-9 (*p* = 0.0456) and MedDRA (*p* = 0.0491), and borderline for the WHO Toxicity Grading Scale (*p* = 0.0587), with broader use of standardized tools in European regions.

### 3.7 Access to external resources supporting the MS-centers

Access to external services supporting MS-Centers (*n* = 168) operations showed regional variation but remained broadly similar between academic and non-academic hospitals (Supplementary Figure S7). The services most frequently available were neurophysiology units, hematology (including support for ASCT), immunology laboratories, and rehabilitation facilities.

In Western Europe, non-academic hospitals generally reported higher rates of access to external services compared to academic centers, suggesting enhanced integration within hospital networks. By contrast, limited access was observed for both academic and non-academic MS-Centers in the Rest of the World and North America. In Other European Countries and Latin America, access levels were intermediate, with no clear pattern favoring one institutional category. A chi-square test of the 10 most reported external services did not reveal significant differences between academic and non-academic institutions (*χ*^2^ = 2.57, df = 8, *p* = 0.96). These findings underline that regional health system factors, rather than academic status, principally determine external resource access.

### 3.8 Communication systems in MS-centers

The implementation of communication systems in MS-Centers (*n* = 168) was generally comparable between academic and non-academic hospitals (Supplementary Table S14, Supplementary Figure S8). Telephone and email were the most widely used tools, consistently identified as the leading channels of communication across all regions and types of institutions. Alternative options (including WhatsApp, mobile apps, and patient portals web pages) were more widespread in academic MS-Centers, particularly in Western Europe and Other European Countries, although these differences did not reach statistical significance. Less common methods (including SMS, web-based platforms, and video calls), showed inconsistent usage, with no distinct patterns by geography or institution type.

A chi-square analysis of the 10 most often reported communication systems found no significant differences between academic and non-academic centers (*χ*^2^ = 18.53, df = 18, *p* = 0.421). These results indicate that conventional communication methods continue to dominate internationally, with digital innovations only modestly integrated into standard MS care practices.

### 3.9 Compliance with MSCU criteria

A simplified classification approach was applied to assess compliance with core MSCU standards, focusing exclusively on academic and non-academic hospitals from Western Europe, Other European Countries, and Latin America. The analysis included only those units meeting at least 70% of high-quality criteria and classified them into two groups: MSCU-I (≥80% compliance) and MSCU-II (70–79% compliance). This classification system used a limited set of high-priority structural and operational indicators, chosen for their high response rates, clinical relevance, and practical value for benchmarking. Key criteria included: MS patient number, staffing levels of neurologists and nurses, access to key diagnostic technologies and therapies, infrastructure components (e.g., day hospitals or biobanks), and core data system functionality (Supplementary Tables S15a-f). Units achieving ≥80% compliance with these requirements (thresholds established in advance by the lead investigators [PS; OF]) were designated as MSCU-I, reflecting fully integrated multidisciplinary care. Those meeting 70–79% were assigned to MSCU-II, indicative of a functional though partly developed model.

In total, 141 MS-Centers met the inclusion criteria. The initial classification of MSCUs into type I (*n* = 120) and type II (*n* = 21) was based on a proportional scoring approach, where units fulfilling ≥80% or 70–79% of 22 predefined criteria were classified as MSCU-I and MSCU-II, respectively, following the methodology agreed by project leaders, as mentioned above. Subsequently, we developed a stricter and more reproducible automatic scoring system based on 16 essential criteria (Supplementary Tables S15e–f), including minimum thresholds for patient volume and staffing. This refined tool may result in different classification outcomes and was used for secondary and sensitivity analyses. Both systems are presented for transparency and to reflect complementary approaches to MSCU benchmarking.

Western Europe had the greatest proportion of MSCU-I units, regardless of institutional type. Latin America, by contrast, showed more heterogeneity, with a higher proportion of MSCU-II centers. Additionally, 5 units serving 500–999 patients annually, with at least one neurologist and one MS nurse, were identified as candidates for a potential MSCU-III category.

Institutional stratification showed that academic hospitals had a significantly higher share of MSCU-I designations than non-academic settings (*χ*^2^ = 6.17, *p* = 0.0458). Likewise, regional analysis indicated a significant association between location and classification status (*χ*^2^ = 22.76, *p* = 0.0001), with Western European MSCUs showing the strongest compliance.

**Figure 5:**
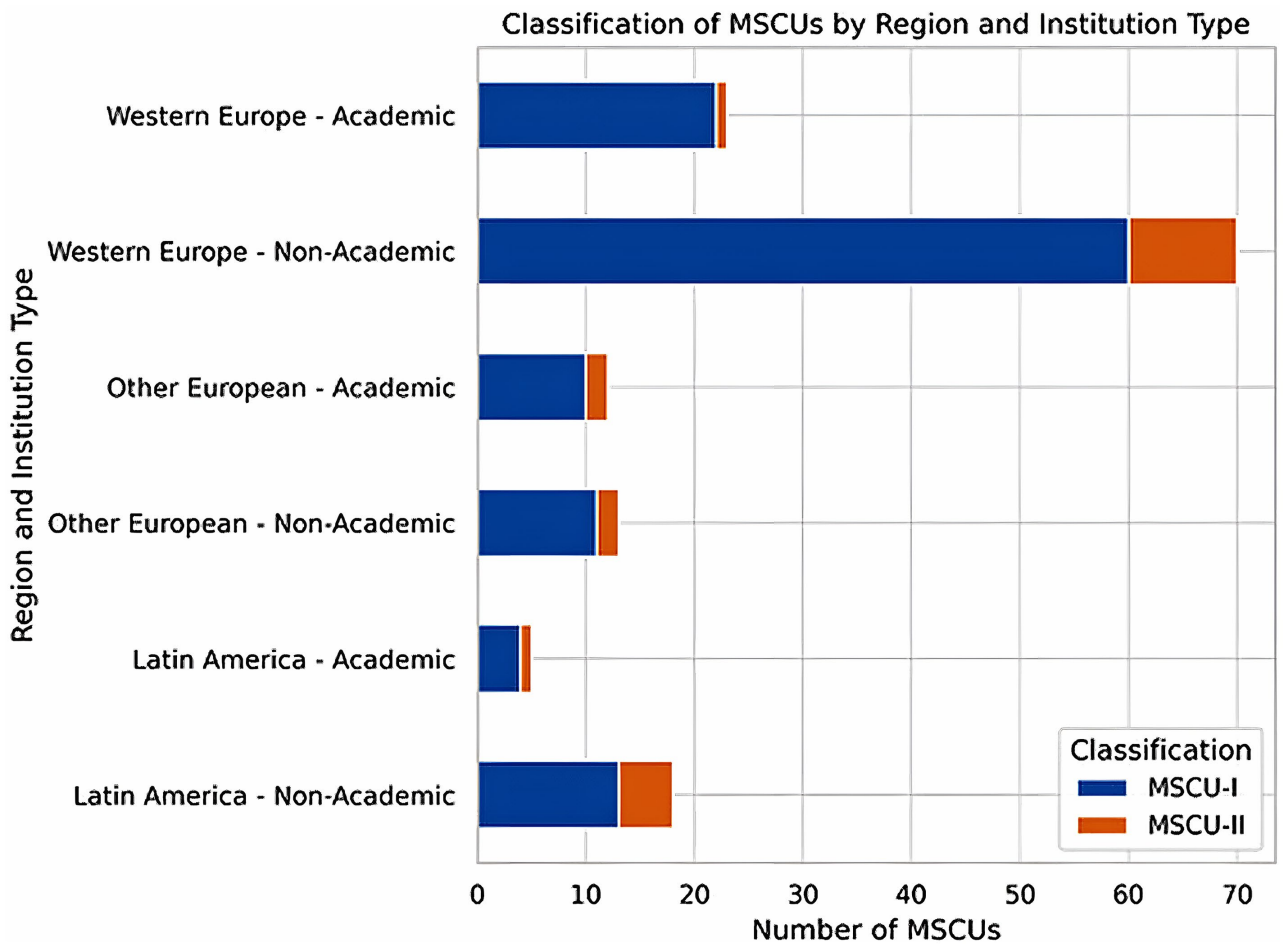
MS-centers compliance by region and hospital type (*n* = 168).

Table 4 details the absolute numbers of MSCU-I and MSCU-II units by region and hospital type, and Figure 5 displays their proportional distributions across the three regions. Overall, these results support a model for MSCU classification that is both regionally adapted and structurally robust, facilitating accurate benchmarking while accounting for local resource differences.

**Table 4:** MSCU compliance by region and hospital type (*n* = 168).

Region	MSCU-I (*n*)		MSCU-II (*n*)	
	Academic hospital	Non-academic hospital	Academic hospital	Non-academic hospital
Western Europe	22	60	1	10
Other European Countries	10	11	2	2
Latin America	4	13	1	5
Total	36	84	4	17

MSCU, multiple sclerosis care units.

### 3.10 Implementation of patient satisfaction and patient-reported outcomes (PROs)

#### 3.10.1 Patient satisfaction

Patient satisfaction assessments were conducted in most MS-Centers (*n* = 168), although marked regional variation was observed (Supplementary Table S16, Supplementary Figure S9). Western Europe reported the highest rates of assessments use (81.3% in academic and 75.7% in non-academic hospitals). In Latin America, the implementation was lower (46.2% for academic and 55.6% for non-academic centers), and the rates were lowest in the Rest of the World (33.3% academic; 50.0% non-academic). However, statistical analysis found no significant differences by region or institution (*χ*^2^ = 1.33, *p* = 0.856), suggesting a widespread effort to measure satisfaction regardless of regional resources.

#### 3.10.2 Patient-reported outcomes (PROs)

The use of PRO measures showed considerable variability across different regions and hospital types (*n* = 168) (Supplementary Tables S17–S18, Supplementary Figure S10). The most widely adopted PROs included quality of life (QoL) assessments, followed by anxiety/depression and fatigue scales. QoL instruments were most frequent in North America and Western Europe, where more than 40% of the centers reported their use.

Academic centers exhibited slightly higher adoption of validated instruments for assessing psychological and fatigue-related symptoms, particularly in Other European Countries and Latin America. By contrast, non-academic facilities more frequently used non-standardized tools. Although differences by institution type were not statistically significant (*p* > 0.05), regional contrasts were evident. For example, up to 33% of academic MS-Centers in Other European Countries used anxiety/depression scales, compared with fewer than 15% in Latin America.

These results point to an unequal integration of structured PRO measures in clinical settings worldwide, and highlight the need for broader, standardized adoption to support comprehensive, patient-centered care.

### 3.11 Economic context and MSCU classification

MSCU-I units were predominantly located in countries with a substantially higher mean Gross Domestic Product (GDP) per capita ($32,466 versus $12,802) and higher health expenditure per capita ($3,639 versus $1,568) than MSCU-II units. Logistic regression analysis demonstrated that both economic indicators were significantly linked to MSCU-I classification, remaining robust after adjustment for region and academic status (*p* < 0.01) (Supplementary Table S19, Supplementary Figures S11–S12).

## 4 Discussion

The present study provides the first comprehensive worldwide evaluation of MS-Centers, presenting a complete overview of their organization, resources, practices and compliance with evidence-based guidelines. Based on data from 198 validated centers in 38 countries, the results highlight outstanding progress as well as ongoing disparities in multidisciplinary MS care delivery.

### 4.1 Geographic and institutional heterogeneity

Marked heterogeneity was evident in the regional distribution and institutional characteristics of MS-Centers. Western Europe led in representation, structural integration, and adherence to MSCU-I standards, while Latin America and Other European Countries showed more diverse patterns. The distribution of academic and non-academic hospitals was unequal, with academic centers being more numerous in Other European Countries, but not in Western Europe or Latin America. This organizational diversity highlights the importance of considering regional health system structures when developing MS care models.

### 4.2 Patient volume and staffing patterns

Patient volume, serving as an indicator of clinical capacity and expertise, was notably higher in academic MS-Centers within Western Europe, followed by similar institutions in Other European Countries and Latin America. Non-academic hospitals also reported large patient volumes, particularly in Western Europe. This challenges the assumption that large-scale care is confined to academic centers and indicates that many non-academic sites function as key referral centers (10–12).

Staffing analyses consistently showed that academic MS-Centers employed more neurologists, nurses and supporting health professionals, especially in Western Europe. Nonetheless, considerable variation within regions, most notably in Latin America, demonstrates that workforce size is influenced by each institution’s mission, funding structure, and national training policies. Although academic centers tended to have larger teams, reflecting their broader roles, no statistically significant interaction between region and institution type emerged.

### 4.3 Academic and professional engagement

Academic centers were more frequently involved in teaching, research and publication. Western European academic MS-Centers reported the highest rates of publication, indicative of well-established research environments. Notably, involvement in teaching and CME was nearly universal across all regions and institution types, highlighting widespread recognition of the importance of ongoing professional development. However, reduced research productivity in Latin American and non-academic centers suggests that structural and funding limitations continue to restrict scientific engagement.

### 4.4 Access to diagnostic and therapeutic resources

#### 4.4.1 Diagnostic resources

The distribution of key diagnostic tools, including blood tests, CSF analysis, EPs, MRI and OCT, showed pronounced variation by region and institutional category. While MRI and blood tests were broadly accessible, their presence was not universal, particularly within certain non-academic settings. Western Europe consistently demonstrated high availability for all major diagnostic techniques, highlighting the impact of established infrastructure and likely reflecting standardized care protocols. In contrast, North America and Latin America showed more variable patterns, with some academic centers unexpectedly reporting limited access to specific tools, such as EPs and OCT.

Notably, these observations challenge the widespread acceptance that academic institutions always offer superior diagnostic resources. In this sense, non-academic centers in Western Europe reported access to diagnostic resources equivalent to that of their academic counterparts. Furthermore, in Other European Countries, the availability of EPs was significantly lower in non-academic (60%) versus academic centers (100%)—a difference confirmed by Fisher’s exact test (*p* = 0.003). A similar trend was seen for blood test availability in Western Europe, where institutional differences reached statistical significance (*p* = 0.012), highlighting notable heterogeneity even within well-resourced environments.

These results underline the necessity for more specific approaches to ensure fair and consistent access to fundamental diagnostic modalities across all institutional and regional contexts. Special attention should be given to expanding the availability of EPs and OCT in under-resourced and inconsistently equipped MS-Centers, as gaps in diagnostic capacity may hinder timely diagnosis and disease monitoring (13).

#### 4.4.2 Therapeutic resources

Although DMTs were widely available in MS-Centers, notable regional differences remained, especially in access to moderate- and high-efficacy treatments. The results indicate that regional healthcare systems, rather than academic status, are the main factor influencing therapeutic availability.

Western Europe and North America exhibited the highest rates of high-efficacy DMT use compared to centers located in Latin America and the Rest of the World. The access and use of high-efficacy DMTs is of high importance as it has consistently been shown that initial high-efficacy treatment compared to low or moderately effective DMTs leads to fewer relapses and disease worsening over time (14), and in patients with disease activity on a low or moderately effective DMT treatment escalation leads to fewer relapses compared with switching to another low or moderately effective therapy (15).

. The variability of ASCT adoption in well-resourced areas like Western Europe suggests that factors such as national policies, reimbursement structures, and logistical considerations play a substantial role, highlighting the role of national regulations, reimbursement models, and logistical barriers (16). Overcoming these challenges will be crucial to ensure fair access to treatment options for all patients with MS (17).

### 4.5 Infrastructure and operational capacity

Our operational analyses (*n* = 168; see Supplementary Table S13) show a higher clinical workload in academic hospitals—reflected by greater annual numbers of treatments and treated relapses, while documentation practices were mixed. Use of administrative data (81.5% overall) and MS registries (85.7%) did not differ by institution type, and the Minimal Data Set (MDS) elements showed similarly high uptake without institutional gaps. In contrast, regional context clearly shaped operations: Western Europe had the highest treatment activity and greater adoption of administrative data systems and registries, whereas Latin America showed lower rates, and cost-accountability was least frequent (20.6%).

These gradients align with broader cross-country differences in MS resources and health-system capacity reported by international datasets and economic indicators (18). Prior work has shown that macro-level resources (GDP, per-capita health spending) are associated with MS service availability and penetration of disease-modifying therapies and registries; our findings refine this picture by demonstrating that, even within hospital settings, region outweighs academic status as a determinant of operational maturity and data-system penetration.

Hospitalization and discharge documentation were heterogeneous. Admission date, discharge date, and hospitalization type were recorded by 67.9, 56.0 and 55.4% of the centers, respectively, with no institutional differences, but with modest regional effects for discharge date and hospitalization type; urgent and programmed admissions showed no regional or institutional differences. Discharge outcomes also varied regionally, with death and inter-hospital transfer being more frequently documented in Western Europe than in Latin America or Other European Countries. Routine recording of visit dates showed a regional effect (higher in Western Europe), while documentation of scheduled and urgent visits did not differ across strata; the most frequent reasons for visit were relapse management, DMT initiation/monitoring (with a regional signal), and diagnostic assessment. The adoption of adverse event classification (ICD-9, WHO Toxicity Grading Scale, MedDRA) was uneven and driven by region rather than institution. Altogether, these results point to region-dependent implementation gaps in information systems and coding standards that are amenable to targeted capacity-building.

### 4.6 Communication systems and access to external services

Communication systems continue to rely on traditional methods, with telephone and email being the most dominant. Academic centers, particularly in Western Europe, more frequently explore digital communication tools, although overall acceptance is still variable. Similarly, access to external services like immunology, neurophysiology, and rehabilitation was shaped more by regional factors than by whether a center was academic or non-academic, emphasizing the foundational role of regional health structures in supporting multidisciplinary care.

### 4.7 Compliance with MSCU-I and MSCU-II standards

The simplified classification model, focused on key structural and operational indicators, provided a practical means of identifying functional MSCUs. Out of 168 centers evaluated, 120 (71.4%) met MSCU-I criteria and 21 (12.5%) met MSCU-II criteria, while 27 (16.1%) did not meet the MSCU standards. Western Europe had the highest number and proportion of MSCU-I units, whereas Latin America contributed the greatest proportion of MSCU-II centers. Academic hospitals were significantly more likely to qualify as MSCU-I (*p* = 0.0458), but regional location proved to be an even stronger predictor (*p* = 0.0001). These outcomes affirm both the robustness and broad applicability of the simplified MSCU-I/MSCU-II classification model.

A main achievement of this study is the optimization of the MSCU classification system using a systematic reduction of the initial 22 criteria. By stepwise analysis, a common set of 16 core requirements was established to reliably categorize MSCUs, regardless of their level of complexity (Type I or II).

This result challenges the assumption that less complex MSCUs (MSCU-II) demand significantly fewer essential elements. Instead, it appears that both categories share a fundamental set of components, with differences in the extent of fulfillment as reflected in the lower thresholds set for MSCU-II units. Notably, criteria such as fluid manipulation, biobanking, and cost or administrative services were consistently found to be non-essential, positioning them as quality enhancements rather than fundamental requirements.

The adoption of a standardized scoring methodology enabled more objective and replicable MSCU classification, reducing subjectivity and adapting variability of data contexts (10, 19). By setting quantitative thresholds for key parameters, this system increases transparency and supports meaningful benchmarking for future audits or health policy evaluations. The lower thresholds for MSCU-II recognize the need for flexibility while still maintaining a structured approach to identifying partially developed care units. Importantly, while the original classification was based on proportional criteria, the automatic scoring model adds a layer of stringency and standardization, enabling consistent application in future benchmarking efforts.

Currently, the MSCU-I/II model may miss high-performance units in regions with smaller populations or decentralized systems. The identification of five centers managing 500–999 patients/year with basic staffing highlights the potential importance of introducing an MSCU-III category to further promote equity and global relevance in MSCU benchmarking. Such a category could describe pragmatic, entry-level MS units that provide core specialized care-typically at least one neurologist and one dedicated MS nurse, regular access to MRI and essential DMTs, and the ability to manage routine follow-up and relapses-while lacking the broader multidisciplinary staffing, advanced subspecialty access, research activity, or infrastructure expected for MSCU-II and MSCU-I. We believe this concept may be particularly relevant for esource-constrained or geographically decentralized settings and deserves prospective validation in future benchmarking studies.

From a policy perspective, the findings advocate for the development of focused accreditation models that emphasize core indicators while allowing adaptation regarding supplementary resources. They also illustrate the importance of pragmatic assessment tools that balance rigor with practicality, especially in settings with limited resources or developing MS services (20).

### 4.8 Patient-centered care: satisfaction and PROs

Although patient-centered care is increasingly prioritized, the systematic evaluation of patient satisfaction and PROs remains inconsistently implemented (21). Western Europe demonstrated comparatively strong adoption, whereas Latin America and other regions reported lower implementation rates. Notably, the institutional type did not significantly influence application, indicating that broader system-level factors and priorities are the primary obstacles. Expanding the routine use of validated instruments, especially those assessing QoL, fatigue, and psychological well-being, will be crucial for ensuring that clinical practice reflects patient experience (22, 23).

### 4.9 Economic context and MSCU classification

Our results highlight the significant impact of national economic resources on the accessibility and structure of high-standard MS care. MSCU-I units were predominantly found in countries with higher GDP and increased health spending, demonstrating how strong economic conditions facilitate advanced infrastructure and staffing. Nonetheless, the existence of MSCU-II units in lower-income countries shows that thoughtful system organization and focused priorities can still support effective MS care, even with limited financial resources (13).

### 4.10 Implications for MSCU policy and certification

Collectively, our results support moving away from purely academic or idealized definitions of MSCUs in favor of adaptable, regionally adapted standards based on measurable criteria. The simplified MSCU-I/MSCU-II model provides a pragmatic certification tool suitable for application within diverse health systems. It balances recognition of both high performance and variation, enabling meaningful comparison while incorporating differing resource levels. While the simplified MSCU-I/MSCU-II approach may not reflect every aspect of multidisciplinary care, it maintains strong discriminative ability, broad feasibility, and practical value for global international benchmarking and policy development.

### 4.11 Strengths and limitations

This study has several strengths, including its large cohort, broad international representation, and the application of a standardized, expert-validated questionnaire. Including both academic and non-academic centers provided a comprehensive perspective on global MS care models. A notable advancement is the creation of a simplified MSCU-I/MSCU-II classification system, based on high-priority indicators with high response rates and clinical significance. This methodology improves applicability across different settings and facilitates scalable benchmarking and policy adoption.

However, certain limitations should be noted. First, the study relied on self-reported data and is therefore vulnerable to reporting bias. Second, the simplified classification framework does not because of missing data or response variability. Third, our grouping of countries into five macro-regions necessarily masks substantial intra-regional heterogeneity in health systems, resources, and care models; readers interested in country-level distribution may consult Supplementary Table S1.

Finally, because participation from North America (*n* = 5) and the Rest of the World (*n* = 6) was limited, the core findings are driven predominantly by data from Europe and Latin America, and generalization to under-represented regions should be made with caution. Future studies should expand participation from these regions and assess how MSCU structural features relate to long-term clinical outcomes.

## 5 Conclusion

Regional context is the primary driver of MSCU performance and standard adherence. Western European centers consistently outperform those in Latin America and Other European Countries across staffing, diagnostics, treatment access, and operational data systems, whereas academic status exerts a smaller, domain-specific effect. Operationally (*n* = 168), academic hospitals delivered more treatments and treated more relapses, but documentation metrics (administrative data, MDS items, registries) showed no institutional gaps, with clear regional gradients instead, higher adoption in Western Europe and lower, more variable uptake elsewhere. Cost-accountability remains limited overall and particularly scarce in Latin America.

A pragmatic reduction from 22 to 16 essential criteria supports a scalable MSCU-I/MSCU-II framework grounded in core structural and functional indicators. This model maintains discriminative power across diverse settings and can underpin benchmarking and certification while acknowledging resource heterogeneity. Introducing a potential MSCU-III category for high-functioning, mid-volume units could broaden inclusivity in constrained environments; pragmatically, such units might be defined by mid-range patient volume, at least one neurologist and one dedicated MS nurse, essential MRI access, and core follow-up/relapse management capacity, but without the broader multidisciplinary or research infrastructure expected for higher-level units. Because the dataset is dominated by centres from Europe and Latin America, extrapolation to under-represented regions should remain cautious. Finally, macro-economic indicators (GDP, health expenditure) remain major correlates of MSCU capability; nonetheless, focused investment in data systems, diagnostic resources (e.g., EPs, OCT), and equitable access to high-efficacy therapies can materially improve performance independent of academic affiliation.

## Data Availability

Access to the data supporting the conclusions of this article may be granted by the authors upon reasonable request, provided that the request is consistent with applicable ethical, legal, institutional, and confidentiality obligations.
