# Supplementary material for: Global overview of multiple sclerosis care units: an international cross-sectional survey
Source: Front Neurol. 2026 Jun 3;17:1812690. doi: 10.3389/fneur.2026.1812690 (PMC13274623; doi:10.3389/fneur.2026.1812690)

# Supplementary Material

## ANNEX I: MSCU QUESTIONNAIRE

General information
- Name of the organization (Hospital/University/Centre)
- Country [selection window]
- Indicate the type of organization There must be an explanation on this point
    - Healthcare organization
    - Hospital
    - Regional authority
    - Research centre
    - Private practitioner

MS in your country
Epidemiology / who gets MS?
- Prevalence rates per 100.000 [Possible responses to select 1 from predefined list:]
    a. <25
    b. 25-50
    c. 51-100
    d. 101-150
    e. 151-200
    f. >200
- Incidence rates per 100.000 [Possible responses to select 1 from predefined list:]
    a. < 1
    b. 1-2
    c. 3-5
    d. 6-10
    e. >10

In your country which proportion of MS patients are cared for?
- MS clinics in University Hospitals [Possible responses to select 1 from predefined list:]
    - < 10%
    - 10-25%
    - 25-50%
    - 50-75%
- MS clinics in Non-academic Hospitals [Possible responses to select 1 from predefined list:]
    - < 10%
    - 10-25%
    - 25-50%
    - 50-75%
- Non-academic hospitals without specific MS clinic [Possible responses to select 1 from predefined list:]
    - < 10%
    - 10-25%
    - 25-50%
    - 50-75%
- Practising Neurologists [Possible responses to select 1 from predefined list: ] Eliminate from the general analysis. Not UNITS
    - < 10%
    - 10-25%
    - 25-50%
    - 50-75%

MS care experience
- Number of patients of MS attended in one year
    - < 500
    - 500-1000
    - 1000-1500
    - 1500-2000
    - 2000-2500
    - 2500-3000
    - 3000-3500
    - 3500-4000
    - 4000-4500
    - 4500-5000

- Processes in MS patients: (access to, routinely used) – This is important. We should know the completeness of the so call units in every geographic area.
  - CSF studies (cerebrospinal fluid)
  - MRI
  - Evoked potentials (EP)
  - OCT (optic coherence tomography)
  - Blood tests

Other activities carried out in your centre
Possible responses to select from and then provide also the quantitative measure. This is important. We should know the completeness of the so call units in every geographic area.

- Teaching [% of overall working activity]
- Continuous Medical Education [% of overall working activity]
- Research [% of overall working activity]
- Publications [Number of publications/year]
- Is previous experience needed when entering the unit
  a. Yes
  b. No

MSCU specific resources
Health care professionals needed for the adequate assistance of MS patients. This is important. We should know the completeness of the so call units in every geographic area.
Possible responses to select from and then provide also the quantitative measure.

- Neurologists [number of Neurologists]
- Nurses [number of Nurses]
- Physiotherapists [number of Physiotherapists]
- Additional health care professionals [Possible responses to select from:] This is important. We should know the completeness of the so call units in every geographic area.
  - Occupational therapist
  - Neuropsychologist
  - Speech therapist
  - Clinical psychologists
  - Rehabilitation neurologist-physician

MSCR specific equipment This is important. We should know the completeness of the so call units in every geographic area.

- Day hospital integrated in the MSCU or available at the centre [Possible responses to select from:]
  - None
  - Yes
  - No
- Area for extraction and manipulation of body fluids (blood, CSF) [Possible responses to select from:]
  - None
  - Yes
  - No
- Area of outpatient assistance [Possible responses to select from:]
  - None
  - Yes
  - No
- System of communications with patients [Possible responses to select from:]
  - E-mail
  - Telephone
  - Mobile (SMS)
  - Web page
  - Other...
- Banking facilities for Sera/CSF [Possible responses to select from:]
  - Yes
  - No
- Resources of other units or services apart from the MSCU, necessary for the adequate provision of care for MS patients [Possible responses to select from:]
  - Service/Unit of Neuroradiology with MRI 1.5 tesla or more
  - Service/Unit of Neurophysiology, with the possibility to perform EP (visual, brainstem, somatosensory and motor)
  - Service/Unit of Nuclear Medicine, with SPECT or PET available, needed in some cases for differential diagnosis
  - Service/Unit of Immunology that can perform IgG oligoclonal bands
  - Service/Unit of Immunology that can perform Neurofilament Light in blood (Simoa)
  - Service/Unit of Rehabilitation with experience in the management of MS patients
  - Service/Unit of Ophthalmology, able to perform OCT and with experience in the management of MS patients

- Service/Unit of Urology, with experience in the management of MS patients
- Service/Unit of Genetics, with possibility for banking of DNA/RNA samples
- Availability of a Haematology Unit for autologous hematopoietic stem cell transplantation (aHSCT)

Indicators of processes and clinical results of the MSCU/year

- Number of treatments administered in the day hospital/outpatient clinic. (Oral or IV) [Possible responses to select from:]
  - 0-200
  - 200-400
  - 400-600
  - 600-800
  - 800-1000
  - 1000-1200
  - 1200-1400
  - 1400-1600
  - 1600-1800
  - 1800-2000
  - 2000 -2200
  - 2200-2400
  - 2400-2600
  - 2600-2800
  - 2800-3000
- Number of treated exacerbations (relapses) [Possible responses to select from:]
  - 0-50
  - 50-100
  - 100-150
  - 150-200
  - 200-250
  - 250-300
  - 300-350
  - 350-400
  - 400-450
  - 450-500

- Number of severe adverse events [Possible responses to select from:]

  - 0-50
  - 50-100
  - 100-150
  - 150-200
- Use of disability progression, expanded disability status scale (EDSS) [Possible responses to select from:]
  - Yes
  - No
- Assessment of degree of satisfaction of users [Possible responses to select from:]
  - Yes
  - No
- Administrative data [Possible responses to select from:]
  - Yes
  - No
- Do you have cost accountability? [Possible responses to select from:]
  - Yes
  - No
  -

MSCU registry

- Does MSCU have a registry of patients with MS? [Possible responses to select from:]
  - None
  - Yes
  - No

[If Yes] Minimal data set requirement - data type collected [Possible responses to select from:]

i. Number of patient identification
ii. Date of birth
iii. Sex
iv. Autonomous community-state -province of residence
v. Date of diagnosis and date of exacerbations (relapses)
vi. Other diagnosis (icd-9-mc)

vii. Diagnostic procedures used in the patient (ICD-9-MC) types of procedures and date of same [Possible responses to select from:]
  a) Clinical assessment [Possible responses to select from:]
    a. EDSS
    b. MSFC
    c. Nhpt
    d. T25FW
    e. SDMT
    f. Cognition
    g. Neuropsychological tests
    h. BICAMS
  b) Patient Reported Outcome assessment [Possible responses to select from:]
    a. Qol MEASURE
    b. FATIGUE: MODIFIED FATIGUE IMPACT SCALE OR OTHER
    c. ANXIETY/DEPRESSION: HOSPITAL ANXIETY AND DEPRESSION SCALE
    d. Other
  c) Blood tests [Possible responses to select from:]
    a. AQ4A
    b. NfL
    c. Immunology serological profile
  d) CSF studies (cerebrospinal fluid) [Possible responses to select from:]
    a. IgG OB
    b. IgM OB
    c. IT Synthesis of IgG
  e) Evoked potentials (EP) [Possible responses to select from:]
    a. Visual EP
    b. Somatosensory EP
    c. Auditory EP
    d. Motor EP

Hospital admissions [Possible responses to select from:]

a) Date of hospitalization
b) Date hospital discharge
c) Type of hospitalization
  - Urgent
  - Programmed
d) Type of hospital discharge
e) MRI
  - Yes
  - No
f) OCT (optic coherence tomography)
  - Yes
  - No
g) X-Ray
  - Yes
  - No
h) Date of visit
i) Type of visit
j) Reason for visit
  - Relapse
  - Initiation or monitoring of disease modifying therapy
  - Diagnostic
  - Follow up
  - Regular annual follow-up in patients not treated with DMTs
  - Infusion
  - MRI
  - Differential Diagnosis

k) Clinical assessment
l) Patient reported outcome assessment
m) Blood tests
n) CSF studies (cerebrospinal fluid)

Treatments available

- Immunomodulator treatment
- Immunosuppressant treatment

- Autologous hematopoietic stem cell transplantation (ASCT)
- Other therapeutic procedures
    - Please specify
- Adverse effects

# ANNEX II

## I. Definition of the needed requirements to be designated as a complete multidisciplinary MSCU (MSCU-I)

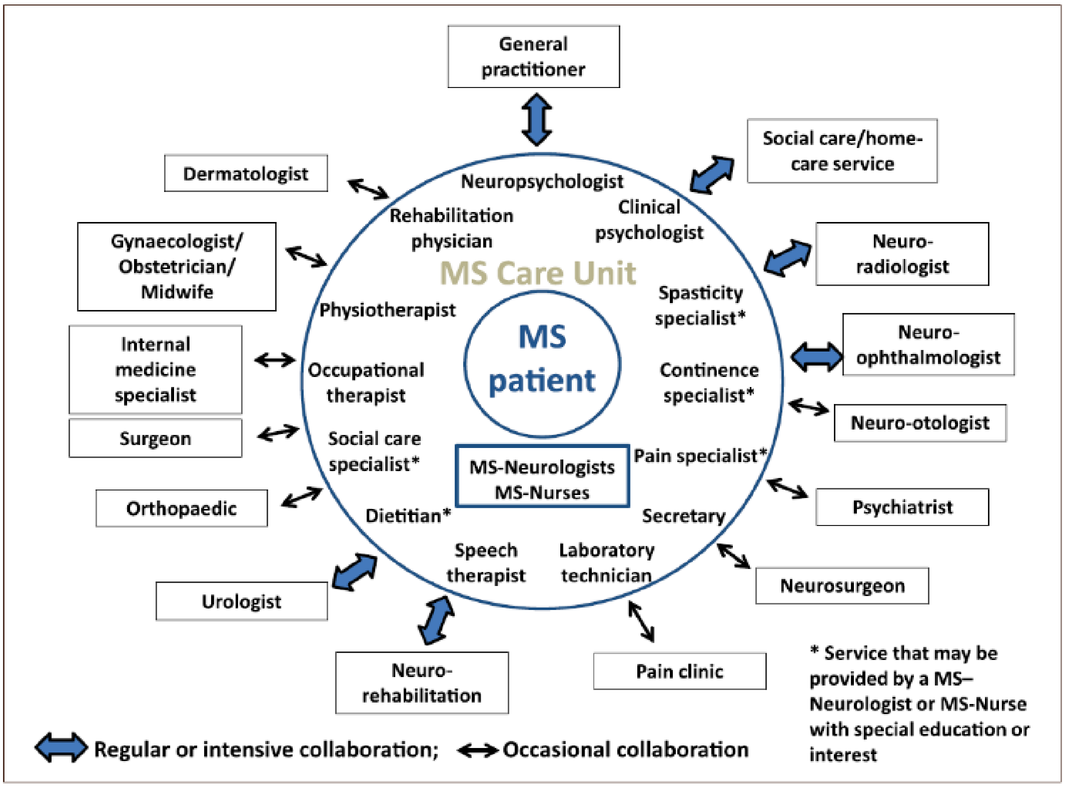


❖ **ACTIVITY:**

- **Number of patients of MS who must be attended per year to ensure adequate care:**
    - 1,000-2,000 patients (new and revisions) per year, of these at least 25-30 new patients/year.
- **Number of procedures that must be performed per year:**

**- Similar to those for which the designation is requested.**
**- Procedures in patients with multiple sclerosis:**

- 100-200 Lumbar punctures/cerebrospinal fluid.
- 500-1,000 MRIs
- **Other data needed on activity:**
- Teaching: Accredited postgraduate teaching: participation of the Unit in the Center's specialist training program. Yes/no (Yes to qualify)
- Participation in Research projects Yes/no (Yes to qualify)
- Publications in the field of MS. (>10/year)
- Continous Medical Education
- Continous training program standardized and authorized by the center's management. Yes/no (Yes to qualify)
- Multidisciplinary clinical sessions, at least monthly, for clinical decision making and treatment coordination). Yes/no (Yes to qualify)

❖ **MSCU SPECIFIC RESOURCES:**

- **Human resources necessary for adequate care of multiple sclerosis patients**
- 3-5 neurologists: Yes/no (Yes to qualify)
- 2-8 Nursing staff: Yes/no (Yes to qualify)
- 1-2 clinical psychologist or daily collaboration: Yes/no (Yes to qualify)
- 1-2 Neuropsychologist or daily collaboration: Yes/no (Yes to qualify)
- 1 Physiotherapist in the MSCU or daily collaboration with physiotherapist: Yes/no (Yes to qualify)
- **Basic training of team members**

- Neurologists with accredited experience of at least 3 years in the care of patients with multiple sclerosis.
- Clinical psychologist accredited with experience of at least 3 years in the care of patients with multiple sclerosis.
- Nursing staff with accredited experience in caring for patients with multiple sclerosis.

❖ **Specific equipment necessary for adequate care of multiple sclerosis.**
- Day hospital integrated into the unit or available in the center, for outpatient IV immunomodulatory/immunosuppressant treatments. Yes/no (Yes to qualify)
- Local for extractions and manipulation of body fluids (blood, CSF). Yes/no (Yes to qualify)
- External consultation exclusive to the unit. Yes/no (Yes to qualify)
- CSF/serum bank: -70ºC, aliquoted, serum and plasma samples. Necessary for immunology, genetic studies, etc. Yes/no (Yes to qualify)
- Communication system with patients, for extraordinary consultations in case of a relapse or complications (telephone, e-mail, website). Yes/no (Yes to qualify)

❖ **ACCESS TO RESOURCES FROM OTHER UNITS OR SERVICES (in addition to those of the MSCU itself are necessary for adequate care of multiple sclerosis)**
- Radiodiagnosis Service/Unit, which has neuroradiology, MRI of 1.5 Tesla or higher. Yes/no (Yes to qualify)
- Neurophysiology Service/Unit, which performs visual, auditory, somatosensory and motor evoked potentials. Yes/no (Yes to qualify)
- Nuclear medicine service/unit, which has SPECT, necessary for differential diagnosis. Yes/no (Yes to qualify)
- Immunology Service/Unit, which performs detection of oligoclonal bands of IgG and/or KFLC index, markers of intrathecal immunoglobulin synthesis that aids in the diagnosis of multiple sclerosis (MS). Routine use of Blood tests (NfL?, AQ4A) . Yes/no (Yes to qualify)
- Rehabilitation Service/Unit with experience in the management of patients with multiple sclerosis. (Physiotherapy, Speech Therapy, Occupational Therapy) Yes/no (Yes to qualify)
- Ophthalmology Service/Unit, which performs optical coherence tomography (OCT) and with experience in the management of patients with multiple sclerosis. Yes/no (Yes to qualify)
- Urology Service/Unit with experience in the management of patients with multiple sclerosis. Yes/no (Yes to qualify)
- Genetics Service/Unit, with the possibility of saving DNA samples for genetic studies. Yes/no (Yes to qualify)
- Hematology Service (AHSCT) Yes/no (Yes to qualify)

Access to all approved DMTs: Yes/no (Yes to qualify)

❖ **PROCEDURE INDICATORS PER YEAR AND CLINICAL RESULTS OF THE MSCU**
- Number of treatments administered in the Day Hospital. >200
- Number of relapses treated > 50
- Disability progression, Expanded Disability Scale (EDSS). Yes/no (Yes to qualify)
- Degree of user satisfaction. Yes/no (Yes to qualify)
- Quality of life measurement (PROs) Yes/no (Yes to qualify)
- Number of complications and their severity: Adverse events, WHO scale: Quantitative variable (number of cases), Qualitative variable (severity). Yes/no (Yes to qualify)

❖ **EXISTENCE OF AN ADEQUATE INFORMATION SYSTEM. (Type of data that the information system must contain to allow knowledge of the activity and evaluation of the quality of the services provided)**
- Completion of the BMDS (basic minimum data set) for hospital discharge in its entirety.
- The Unit must have a record of patients with multiple sclerosis that must include at least: Yes/no (Yes to qualify)
  - Medical History Number.
  - Date of birth.
  - Sex.
  - Address of habitual residence of the patient.
  - Date of admission and date of discharge.
  - Type of admission (Urgent, scheduled, others).
  - Type of discharge (Home, transfer to hospital, voluntary discharge, death, transfer to social and health center, others).
  - Service responsible for patient discharge.
  - Main diagnosis (ICD-9-CM): Date of diagnosis and date of outbreaks.
  - Other diagnoses (ICD-9-CM).
  - Diagnostic procedures performed on the patient (ICD-9-CM): Types of procedures and date of performance.
  - Therapeutic procedures performed on the patient (ICD-9-CM): Types of procedures and dates of performance. Immunomodulatory treatment. Immunosuppressive treatment. Bone marrow transplant. Other therapeutic procedures.

- Complications (ICD-9-CM).
- Follow-up: Number of relapses. Progression of disability,
- QoL evaluation

The unit must have the precise data that it must send to the National Health System for the annual monitoring of the reference unit.

## II. Definition of the minimum requirement to be designated as a multidisciplinary MSCU (MSCU-II)

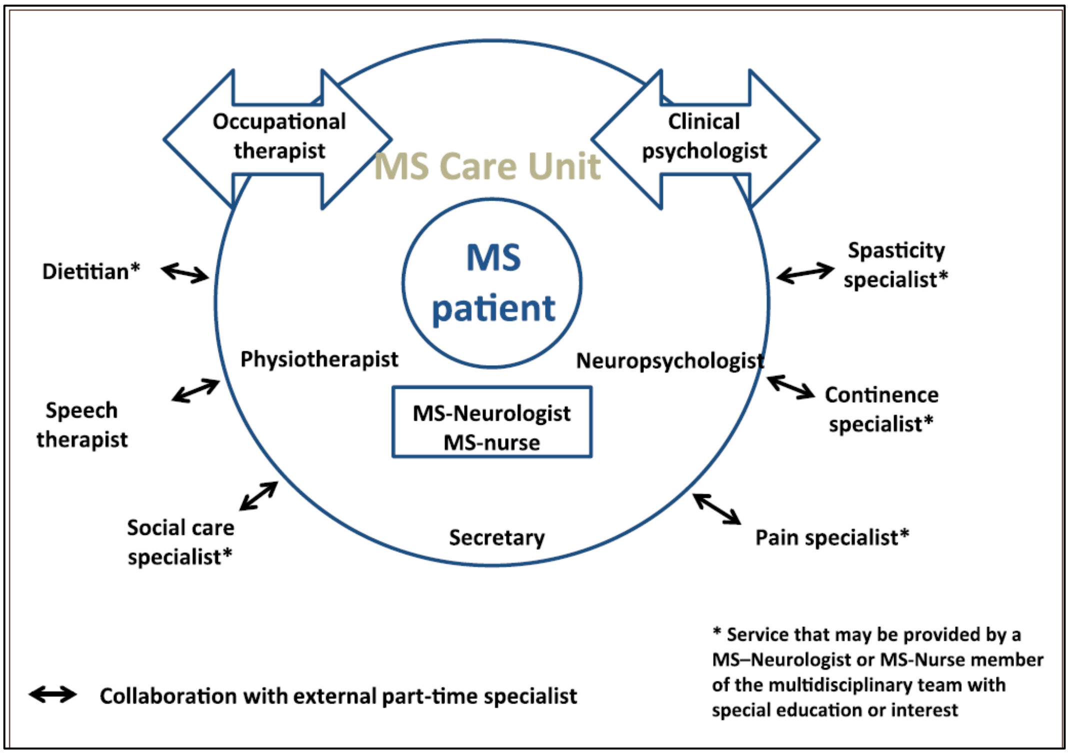


- **ACTIVITY:**
- **Number of patients of MS who must be attended per year to ensure adequate care:**
    - 500-1,000 patients (new and revisions) per year, of these at least 15-20 new patients/year.
- **Number of procedures that must be performed per year:**

**- Similar to those for which the designation is requested.**
**- Procedures in patients with multiple sclerosis:**

- 50-100 Lumbar punctures/cerebrospinal fluid.
- 100-500 MRIs
- **Other data needed on activity:**
- Publications in the field of MS. (>5/year)
- Continous Medical Education
- Continous training program standardized and authorized by the center's management. Yes/no (Yes to qualify)
- Multidisciplinary clinical sessions, at least monthly, for clinical decision making and treatment coordination). Yes/no (Yes to qualify)
-
- **MSCU SPECIFIC RESOURCES:**
- **Human resources necessary for adequate care of multiple sclerosis patients**
- 2-3 neurologists: Yes/no (Yes to qualify)
- 2-4 Nursing staff: Yes/no (Yes to qualify)
- 1 clinical psychologist or daily collaboration: Yes/no (Yes to qualify)
- 1 Neuropsychologist or daily collaboration: Yes/no (Yes to qualify)
- 1 Physiotherapist in the MSCU or daily collaboration with physiotherapist: Yes/no (Yes to qualify)
- **Basic training of team members**
- Neurologists with accredited experience of at least 3 years in the care of patients with multiple sclerosis.
- Clinical psychologist accredited with experience of at least 3 years in the care of patients with multiple sclerosis.
- Nursing staff with accredited experience in caring for patients with multiple sclerosis.
- **Specific equipment necessary for adequate care of multiple sclerosis.**

- Day hospital integrated into the unit or available in the center, for outpatient IV immunomodulatory/immunosuppressant treatments. Yes/no (Yes to qualify)
- Local for extractions and manipulation of body fluids (blood, CSF). Yes/no (Yes to qualify)
- External consultation exclusive to the unit. Yes/no (Yes to qualify)
- Communication system with patients, for extraordinary consultations in case of a relapse or complications (telephone, e-mail, website). Yes/no (Yes to qualify)

❖ **ACCESS TO RESOURCES FROM OTHER UNITS OR SERVICES (in addition to those of the MSCU itself are necessary for adequate care of multiple sclerosis)**

- Radiodiagnosis Service/Unit, which has neuroradiology, MRI of 1.5 Tesla or higher. Yes/no (Yes to qualify)
- Neurophysiology Service/Unit, which performs visual, auditory, somatosensory and motor evoked potentials. Yes/no (Yes to qualify)
- Immunology Service/Unit, which performs detection of oligoclonal bands of IgG and/or KFLC index, markers of intrathecal immunoglobulin synthesis that aids in the diagnosis of multiple sclerosis (MS). Routine use of Blood tests (NfL?, AQ4A) . Yes/no (Yes to qualify)
- Rehabilitation Service/Unit with experience in the management of patients with multiple sclerosis. (Physiotherapy, Speech Therapy, Occupational Therapy) Yes/no (Yes to qualify)
- Ophthalmology Service/Unit, which performs optical coherence tomography (OCT) and with experience in the management of patients with multiple sclerosis. Yes/no (Yes to qualify)
- Urology Service/Unit with experience in the management of patients with multiple sclerosis. Yes/no (Yes to qualify)
- Genetics Service/Unit, with the possibility of saving DNA samples for genetic studies. Yes/no (Yes to qualify)
- Hematology Service (AHSCT) Yes/no (Yes to qualify)
- Access to all approved DMTs: Yes/no (Yes to qualify)

❖ **PROCEDURE INDICATORS PER YEAR AND CLINICAL RESULTS OF THE MSCU**

- Number of treatments administered in the Day Hospital. >50
- Number of relapses treated > 20
- Disability progression, Expanded Disability Scale (EDSS). Yes/no (Yes to qualify)
- Degree of user satisfaction. Yes/no (Yes to qualify)
- Quality of life measurement (PROs) Yes/no (Yes to qualify)
- Number of complications and their severity: Adverse events, WHO scale: Quantitative variable (number of cases), Qualitative variable (severity). Yes/no (Yes to qualify)

❖ **EXISTENCE OF AN ADEQUATE INFORMATION SYSTEM. (Type of data that the information system must contain to allow knowledge of the activity and evaluation of the quality of the services provided)**

- Completion of the BMDS (basic minimum data set) for hospital discharge in its entirety.
- The Unit must have a record of patients with multiple sclerosis that must include at least: Yes/no (Yes to qualify)
  - Medical History Number.
  - Date of birth.
  - Sex.
  - Address of habitual residence of the patient.
  - Date of admission and date of discharge.
  - Type of admission (Urgent, scheduled, others).
  - Type of discharge (Home, transfer to hospital, voluntary discharge, death, transfer to social and health center, others).
  - Service responsible for patient discharge.
  - Main diagnosis (ICD-9-CM): Date of diagnosis and date of outbreaks.
  - Other diagnoses (ICD-9-CM).
  - Diagnostic procedures performed on the patient (ICD-9-CM): Types of procedures and date of performance.
  - Therapeutic procedures performed on the patient (ICD-9-CM): Types of procedures and dates of performance. Immunomodulatory treatment. Immunosuppressive treatment. Bone marrow transplant. Other therapeutic procedures.
  - Complications (ICD-9-CM).
  - Follow-up: Number of relapses. Progression of disability.

## Supplementary Tables


- Table S1. Distribution of Participating MS-Centers by Country (N=198)
- Table S2. Patient Volume per Year Attended by Region and Type of Hospital (N=168).
- Table S3. MS-Center Academic Activities by Region and Hospital Type(N=168).
- Table S4. Availability and utilization of diagnostic resources (Blood tests, CSF studies, evoked potentials, MRI, OCT) across regions and differences by type of hospitals (non-academic and academic hospitals ) (N=168).
- Table S5. Use of Immunomodulatory Treatments by Hospital Type
- Table S6. Use of Immunomodulatory Treatments by Region (N=168)
- Table S7. Use of Immunosuppressant Treatments by Hospital Type (N=168).
- Table S8. Use of Immunosuppressant Treatments by Region
- Table S9. Use of Immunosuppressant Treatments by Region and Hospital Type (N=168).
- Table S10. Generic and Commercial Names of MS Therapies
- Table S11. Distribution of MS Therapy Use by Efficacy Level, Hospital Type, and Region (N=168) (including ASCT Separately).
- Table S12. Availability of Core Structural Components in MS-Centers by Region (N=168) by Region and Hospital Type
- Table S13. Operational Characteristics of MS-Centers (N=198)
- Table S14. Communication Systems Used in MS-Centers by Hospital Type (N=168).
- Table S15a. Essential and Non-Essential Criteria for MSCU Classification
- Table S15b. Summary of MSCU-I / MSCU-II Classification Criteria (1)
- Table S15c. Summary of Final MSCU Classification Criteria of Hospital MS-Centers (N=168) (2)
- Table S15d. Compliance with MSCU Criteria by Hospital Type and Region (N=168).
- Table S15e. Summary Table of Criteria for MSCU-I / MSCU-II Classification with Minimum Thresholds
- Table S15f. Automatic Scoring Scheme for MSCU Classification
- Table S16. Assessment of User Satisfaction by Region and Type of Hospital (N=168).
- Table S17. Distribution of Patient-Reported Outcome Assessments by Region and Hospital Type (absolute number) (N=168).

- Table S18. Distribution of Patient-Reported Outcome Assessments by Region and Hospital Type (%) (N=168).
- Table S19. GDP and Health Expenditure per Capita by MSCU Type

## Table S1: Distribution of Participating MS-Centers per Country (N=198)

| Country | Orgs | Country | Orgs | Country | Orgs | Country | Orgs | Country | Orgs |
|---|---|---|---|---|---|---|---|---|---|
| Argentina | 21 | Colombia | 7 | Honduras | 1 | Paraguay | 2 | Sweden | 3 |
| Australia | 1 | Denmark | 5 | Hungary | 3 | Poland | 10 | Switzerland | 7 |
| Austria | 2 | Estonia | 1 | Israel | 2 | Portugal | 10 | Tunisia | 1 |
| Belgium | 4 | Finland | 4 | Italy | 10 | Romania | 2 | Turkey | 2 |
| Brazil | 3 | France | 25 | Japan | 1 | Russia | 3 | United Kingdom | 4 |
| Bulgaria | 3 | Germany | 5 | Lebanon | 1 | Serbia | 2 | United States | 4 |
| Canada | 5 | Greece | 1 | Lithuania | 3 | Slovenia | 5 | Uruguay | 3 |
| Chile | 9 | Guatemala | 3 | Norway | 5 | Spain | 19 | | |
| TOTAL Countries | 38 | TOTAL Orgs | 198 | | | | | | |

## Table S2. Patient Volume per Year Attended by Region and Type of Hospital (N=168).

| Region | Non-Academic Hospitals (N=124) (Mean ± SD) | Academic Hospitals (N=44) (Mean ± SD) |
|---|---|---|
| Western Europe | 1426 ± 853 | 2228 ± 901 |
| Other European Countries | 1301 ± 603 | 1875 ± 704 |
| North America | 918 ± 329 | 2000 ± 894 |
| Latin America | 860 ± 745 | 1133 ± 854 |
| Rest of World | 772 ± 412 | 1011 ± 320 |

## Table S3. MS-Center Academic Activities by Region and Hospital Type (N=168)

| Region | Type | Teaching _Total | CME _Total | Research _Total | Mean Publications | p-value |
|---|---|---|---|---|---|---|
| Western Europe | Academic | 23 | 23 | 23 | 26.41 | 0.0007 |
| Western Europe | Non-Academic | 75 | 75 | 75 | 14.14 | 0.0007 |
| Other Europe | Academic | 13 | 13 | 13 | 11.73 | 0.1037 |
| Other Europe | Non-Academic | 15 | 15 | 15 | 7.08 | 0.1037 |
| Latin America | Academic | 5 | 5 | 5 | 13.50 | 0.0673 |
| Latin America | Non-Academic | 27 | 27 | 27 | 8.68 | 0.0673 |

## Table S4. Availability and utilization of diagnostic resources (Blood tests, CSF studies, evoked potentials, MRI, OCT) across regions and differences by type of hospitals (Non-Academic and Academic hospitals) (N=168)

| Region | Process | Non-Academic (%) | Academic (%) | Difference (%) | p-value |
|---|---|---|---|---|---|
| Western Europe | BloodTests | 70.7 | 95.7 | -25.0 | 0.012 |
| Western Europe | CSFStudies | 78.7 | 91.3 | -12.6 | 0.227 |
| Western Europe | EvokedPotentials | 76.0 | 91.3 | -15.3 | 0.145 |
| Western Europe | MRI | 90.7 | 95.7 | -5.0 | 0.676 |
| Western Europe | OCT | 90.7 | 95.7 | -5.0 | 0.676 |
| Other European Countries | BloodTests | 66.7 | 69.2 | -2.5 | 1.0 |
| Other European Countries | CSFStudies | 73.3 | 69.2 | 4.1 | 1.0 |
| Other European Countries | EvokedPotentials | 53.3 | 69.2 | -15.9 | 0.46 |
| Other European Countries | MRI | 73.3 | 69.2 | 4.1 | 1.0 |
| Other European Countries | OCT | 73.3 | 69.2 | 4.1 | 1.0 |
| North America | BloodTests | 50.0 | 0.0 | 50.0 | 0.467 |
| North America | CSFStudies | 50.0 | 0.0 | 50.0 | 0.467 |
| North America | EvokedPotentials | 50.0 | 0.0 | 50.0 | 0.467 |
| North America | MRI | 100.0 | 50.0 | 50.0 | 0.333 |
| North America | OCT | 100.0 | 50.0 | 50.0 | 0.333 |
| Latin America | BloodTests | 63.3 | 100.0 | -36.7 | 0.157 |
| Latin America | CSFStudies | 60.0 | 100.0 | -40.0 | 0.141 |
| Latin America | EvokedPotentials | 53.3 | 100.0 | -46.7 | 0.069 |
| Latin America | MRI | 80.0 | 100.0 | -20.0 | 0.561 |
| Latin America | OCT | 80.0 | 100.0 | -20.0 | 0.561 |
| Rest of the World | BloodTests | 33.3 | 50.0 | -16.7 | 1.0 |
| Rest of the World | CSFStudies | 33.3 | 50.0 | -16.7 | 1.0 |
| Rest of the World | EvokedPotentials | 33.3 | 50.0 | -16.7 | 1.0 |
| Rest of the World | MRI | 66.7 | 50.0 | 16.7 | 1.0 |
| Rest of the World | OCT | 66.7 | 50.0 | 16.7 | 1.0 |

## Table S5. Use of Immunomodulatory Treatments by Hospital Type (N=168)

| Treatment | Academic Hospital | Non-Academic Hospital | p-value (Academic vs Non-Academic) |
|---|---|---|---|
| Avonex | 45 | 122 | 1.0 |
| Betaseron | 44 | 107 | 0.1442 |
| Copaxone | 46 | 116 | 0.1952 |
| Extavia | 27 | 71 | 0.9507 |
| Generic Glatiramer | 34 | 87 | 0.7112 |
| Plasmapheresis | 45 | 109 | 0.0993 |
| Plegridy® (PEG-Interferon) | 43 | 108 | 0.3347 |
| Rebif® (interferon beta-1a) | 45 | 124 | 1.0 |
| Vumerity ® (Diroximel fumarate) | 14 | 43 | 0.7686 |

## Table S6. Use of Immunomodulatory Treatments by Region (N=168)

| Treatment | Western Europe | Other European Countries | North America | Latin America | Rest of the World | p-value (Academic vs Non-Academic) |
|---|---|---|---|---|---|---|
| Avonex | 103 | 28 | 9 | 51 | 6 | 1.0 |
| Betaseron | 97 | 29 | 9 | 36 | 6 | 0.1442 |
| Copaxone | 102 | 30 | 9 | 40 | 4 | 0.1952 |
| Extavia | 83 | 14 | 6 | 2 | 0 | 0.9507 |
| Generic Glatiramer | 79 | 18 | 9 | 41 | 3 | 0.7112 |
| Plasmapheresis | 96 | 23 | 7 | 45 | 5 | 0.0993 |
| Plegridy® (PEG-Interferon) | 102 | 28 | 9 | 29 | 5 | 0.3347 |
| Rebif® (interferon beta-1a) | 103 | 29 | 9 | 53 | 5 | 1.0 |
| Vumerity ® (Diroximel fumarate) | 47 | 5 | 5 | 5 | 3 | 0.7686 |

## Table S7. Use of Immunosuppressant Treatments by Hospital Type (N=168)

| Treatment | Academic Hospital | Non-Academic Hospital | p-value (Academic vs Non-Academic) |
|---|---|---|---|
| Aubagio® (teriflunomide) | 46 | 118 | 0.3068 |
| Azathioprine | 38 | 110 | 0.5555 |
| Cyclophosphamide | 33 | 101 | 0.3163 |
| Gilenya® (fingolimod) | 47 | 120 | 0.1782 |
| IgIV | 1 | 1 | 1.0 |
| Kesimpta (ofatumumab) | 1 | 1 | 1.0 |
| Lemtrada® (alemtuzumab) | 39 | 98 | 0.4804 |
| Mavenclad® (cladribine) | 43 | 122 | 0.5496 |
| Methotrexate | 33 | 97 | 0.581 |
| Mycophenolate Mofetil | 0 | 1 | 1.0 |
| Novantrone® (mitoxantrone) | 35 | 87 | 0.5197 |
| Ponesimod (Ponvory) | 1 | 0 | 0.6005 |

## Table S8. Use of Immunosuppressant Treatments by Region (N=168)

| Treatment | Western Europe | Other European Countries | North America | Latin America | Rest of the World | p-value (Academic vs Non-Academic) |
|---|---|---|---|---|---|---|
| Aubagio® (teriflunomide) | 98 | 28 | 6 | 26 | 3 | 0.3068 |
| Azathioprine | 88 | 20 | 4 | 28 | 5 | 0.5555 |
| Cyclophosphamide | 82 | 13 | 5 | 26 | 5 | 0.3163 |
| Gilenya® (fingolimod) | 96 | 27 | 6 | 30 | 5 | 0.1782 |
| IgIV | 1 | 0 | 0 | 0 | 0 | 0.6005 |
| Kesimpta (ofatumumab) | 1 | 0 | 0 | 0 | 1 | 0.6005 |
| Lemtrada® (alemtuzumab) | 72 | 25 | 6 | 28 | 3 | 0.4804 |
| Mavenclad® (cladribine) | 98 | 27 | 6 | 29 | 2 | 0.5496 |
| Mayzent (siponimod) | 0 | 0 | 0 | 0 | 1 | 1.0 |
| Methotrexate | 82 | 12 | 4 | 24 | 5 | 0.581 |
| Mycophenolate Mofetil | 1 | 0 | 1 | 0 | 0 | 1.0 |
| Novantrone® (mitoxantrone) | 82 | 18 | 4 | 14 | 2 | 0.5197 |
| HSCT | 1 | 0 | 0 | 0 | 0 | 1.0 |
| Ponesimod (Ponvory®) | 1 | 0 | 0 | 0 | 0 | 1.0 |
| Rituximab | 95 | 19 | 5 | 35 | 4 | 0.085 |
| Tecfidera® (dimethyl fumarate) | 98 | 27 | 6 | 26 | 5 | 1.0 |
| Tysabri® (natalizumab) | 97 | 28 | 6 | 28 | 5 | 0.5964 |
| Zeposia (Ozanimod®) | 1 | 0 | 0 | 0 | 0 | 0.6005 |
| evobrutinib, fenebrutinib | 0 | 1 | 0 | 0 | 0 | 0.6005 |
| Ocrevus® (ocrelizumab) | 92 | 28 | 6 | 32 | 4 | 0.3836 |

## Table S9. Use of Immunosuppressant Treatments by Region and Hospital Type (N=168)

| Treatment | Western Europe - Academic | Western Europe - Non-Academic | Other European Countries - Academic | Other European Countries - Non-Academic | North America - Academic | North America - Non-Academic | Latin America - Academic | Latin America - Non-Academic | Rest of the World - Academic | Rest of the World - Non-Academic | p-value (Academic vs Non-Academic) |
|---|---|---|---|---|---|---|---|---|---|---|---|
| Aubagio® (teriflunomide) | 23 | 75 | 13 | 15 | 2 | 4 | 5 | 21 | 1 | 2 | 0.3068 |
| Azathioprine | 20 | 68 | 8 | 12 | 1 | 3 | 5 | 23 | 2 | 3 | 0.5555 |
| Cyclophosphamide | 18 | 64 | 4 | 9 | 2 | 3 | 5 | 21 | 2 | 3 | 0.3163 |
| Gilenya® (fingolimod) | 23 | 73 | 13 | 14 | 2 | 4 | 5 | 25 | 2 | 3 | 0.1782 |
| IgIV | 1 | 0 | 0 | 0 | 0 | 0 | 0 | 0 | 0 | 0 | 0.6005 |
| Kesimpta (ofatumumab) | 0 | 1 | 0 | 0 | 0 | 0 | 0 | 0 | 1 | 0 | 1.0 |
| Lemtrada® (alemtuzumab) | 18 | 54 | 12 | 13 | 2 | 4 | 4 | 24 | 1 | 2 | 0.4804 |
| Mavenclad® (cladribine) | 23 | 75 | 12 | 15 | 2 | 4 | 4 | 25 | 0 | 2 | 0.5496 |
| Mayzent (siponimod) | 0 | 0 | 0 | 0 | 0 | 0 | 0 | 0 | 0 | 1 | 1.0 |
| Methotrexate | 19 | 63 | 4 | 8 | 1 | 3 | 5 | 19 | 2 | 3 | 0.581 |
| Mycophenolate Mofetil | 0 | 1 | 0 | 0 | 0 | 1 | 0 | 0 | 0 | 0 | 1.0 |
| Novantrone® (mitoxantrone) | 20 | 62 | 9 | 9 | 1 | 3 | 3 | 11 | 0 | 2 | 0.5197 |
| HSCT | 0 | 1 | 0 | 0 | 0 | 0 | 0 | 0 | 0 | 0 | 1.0 |
| Ponesimod (Ponvory®) | 0 | 1 | 0 | 0 | 0 | 0 | 0 | 0 | 0 | 0 | 1.0 |
| Rituximab | 22 | 73 | 9 | 10 | 1 | 4 | 5 | 30 | 1 | 3 | 0.085 |
| Tecfidera® (dimethyl fumarate) | 23 | 75 | 12 | 15 | 2 | 4 | 3 | 23 | 2 | 3 | 1.0 |
| Tysabri® (natalizumab) | 23 | 74 | 13 | 15 | 2 | 4 | 4 | 24 | 2 | 3 | 0.5964 |
| Zeposia (ozanimod®) | 1 | 0 | 0 | 0 | 0 | 0 | 0 | 0 | 0 | 0 | 0.6005 |
| evobrutinib, fenebrutinib | 0 | 0 | 1 | 0 | 0 | 0 | 0 | 0 | 0 | 0 | 0.6005 |
| Ocrevus® (ocrelizumab) | 23 | 69 | 13 | 15 | 2 | 4 | 5 | 27 | 1 | 3 | 0.3836 |

## Table S10. Generic and Commercial Names of MS Therapies

| Generic Name | Commercial Name |
|---|---|
| Glatiramer acetate | Copaxone |
| Interferón beta-1a | Avonex, Rebif |
| Interferón beta-1b | Betaferon, Betaseron, Extavia |
| Peginterferón beta-1a | Plegridy |
| Teriflunomida | Aubagio |
| Dimetil fumarato | Tecfidera |
| Diroximel fumarato | Vumerity |
| Fingolimod | Gilenya |
| Siponimod | Mayzent |
| Ponesimod | Ponvory |
| Ozanimod | Zeposia |
| Natalizumab | Tysabri |
| Cladribine | Mavenclad |
| Alemtuzumab | Lemtrada |
| Ocrelizumab | Ocrevus |
| Ofatumumab | Kesimpta |
| Mitoxantrone | Novantrone |
| Autologous stem Cell Transplantation | ASCT |

## Table S11. MS Therapy Use by Efficacy Level, Region and Hospital Type (N=168) (Including ASCT Separately)

| Region | Institution Type | Low Efficacy (%) | Moderate Efficacy (%) | High Efficacy (%) | ASCT (%) |
|---|---|---|---|---|---|
| Western Europe | Academic Hospital | 90 | 75 | 116.5 | 56.5 |
| Western Europe | Non-Academic Hospital | 88 | 73 | 119.3 | 61.3 |
| Other European Countries | Academic Hospital | 85 | 70 | 93.5 | 38.5 |
| Other European Countries | Non-Academic Hospital | 83 | 68 | 93.0 | 40.0 |
| North America | Academic Hospital | 88 | 78 | 65.0 | 0.0 |
| North America | Non-Academic Hospital | 86 | 76 | 138.0 | 75.0 |
| Latin America | Academic Hospital | 80 | 68 | 90.0 | 40.0 |
| Latin America | Non-Academic Hospital | 78 | 66 | 59.1 | 11.1 |
| Rest of the World | Academic Hospital | 75 | 60 | 95.0 | 50.0 |
| Rest of the World | Non-Academic Hospital | 73 | 58 | 43.0 | 0.0 |

Note: Percentages by efficacy level are not mutually exclusive. A single MS-Center may report the use of therapies from multiple efficacy categories (e.g., both moderate- and high-efficacy DMTs), and therefore, totals across efficacy levels may exceed 100%.

## Table S12. Availability of Core Structural Components in MS-Centers (N=168) by Region and Hospital Type

| Region_Organization | Biological Fluids Area | Outpatient Area | Banking Facilities | Day Hospital |
|---|---|---|---|---|
| Western European Countries - Academic Hospital | 1.0 | 1.0 | 0.96 | 0.91 |
| Western European Countries - Non-Academic Hospital | 0.95 | 0.95 | 0.83 | 0.92 |
| Other European Countries - Academic Hospital | 0.92 | 1.0 | 0.92 | 1.0 |
| Other European Countries - Non-Academic Hospital | 1.0 | 0.87 | 0.8 | 0.93 |
| North America - Academic Hospital | 0.5 | 1.0 | 0.5 | 0.5 |
| North America - Non-Academic Hospital | 1.0 | 1.0 | 0.5 | 0.75 |
| Latin America - Academic Hospital | 0.8 | 1.0 | 0.6 | 1.0 |
| Latin America - Non-Academic Hospital | 0.87 | 0.9 | 0.53 | 0.63 |
| Rest of the World - Academic Hospital | 1.0 | 1.0 | 1.0 | 1.0 |
| Rest of the World - Non-Academic Hospital | 1.0 | 1.0 | 1.0 | 1.0 |

## Table S13 – Operational Characteristics of MS-Centers (N=168)

| Operational variable | % of centers (N=168) | p (Academic vs Non-Academic) | p (by Region) |
|---|---|---|---|
| Treatments administered (data present) | 100.0 | <0.001 | <0.0001 |
| Treated exacerbations (data present) | 100.0 | <0.0001 | <0.0001 |
| Severe adverse events (data present) | 100.0 | 0.5068 | 0.2005 |
| Administrative data recorded | 81.5 | 0.6034 | 0.0015 |
| Cost accountability | 53.0 | 0.0538 | <0.0001 |
| MS patient registry | 85.7 | 1.0000 | 0.0185 |
| Minimal dataset documented | 100.0 | n.s. | n.s. |
| Hospital admissions data (any subitem) | 100.0 | n.s. | n.s. |
| Type of hospital discharge (any subitem) | 100.0 | n.s. | n.s. |
| Date of visit recorded | 75.0 | 0.6198 | 0.0236 |
| Type of visit recorded | 100.0 | n.s. | n.s. |
| Reason for visit recorded | 100.0 | n.s. | n.s. |
| Adverse effects classification | 100.0 | n.s. | n.s. |

## Table S14. Communication Systems Used in MS-Centers by Hospital Type (N=168)

| Communication System | Academic Hospitals | Non-Academic Hospitals |
|---|---|---|
| Phone | 45 | 123 |
| E-mail | 42 | 108 |
| SMS | 25 | 62 |
| Website | 20 | 26 |
| Patient Portal | 2 | 1 |
| Social Media | 1 | 2 |
| App | 2 | 1 |
| WhatsApp | 0 | 2 |
| Personal | 0 | 1 |
| Personal Interview | 0 | 1 |
| Digital Mail | 0 | 1 |
| Postal | 0 | 1 |
| Mail, Digital Tool | 0 | 1 |
| Secured Healthcare Network | 0 | 1 |
| Mail | 0 | 1 |
| Through The Electronic Patient Record | 0 | 1 |
| Twitter | 1 | 0 |
| Video Call | 1 | 0 |
| Letter | 0 | 1 |

## Table S15a. Essential and Non-Essential Criteria for MSCU Classification

| Criterion | Essential for MSCU-I / MSCU-II | Impact on Classification |
|---|---|---|
| MS patient volume | Yes / Yes | Essential |
| Number of neurologists | Yes / Yes | Essential |
| Number of nurses | Yes / No | Essential for MSCU-I only |
| Presence of physiotherapists | Yes / Yes | Essential |
| Access to MRI | Yes / Yes | Essential |
| Access to immunology services | Yes / Yes | Essential |
| Access to neuropsychology | No / Yes | Essential for MSCU-II only |
| Access to rehabilitation services | Yes / Yes | Essential |
| Continuing medical education (CME) | Yes / No | Essential for MSCU-I only |
| Participation in research | Yes / Yes | Essential |
| Day hospital availability | Yes / Yes | Essential |
| Access to clinical psychology | Yes / Yes | Essential |
| Outpatient consultation area | Yes / Yes | Essential |
| EDSS registry | Yes / Yes | Essential |
| Patient communication system | No / Yes | Essential for MSCU-II only |
| Access to DMTs | Yes / Yes | Essential |
| Area for fluid extraction/handling | No / No | Non-essential |
| Biobanking (sera/CSF) | No / No | Non-essential |
| Administrative data system | No / No | Non-essential |
| Cost accountability system | No / No | Non-essential |
| MS patient registry | Yes / Yes | Essential |
| Assessment of patient satisfaction | Yes / Yes | Essential |

## Table S15b. Summary of MSCU-I / MSCU-II Criteria

| Criterion | Response Rate (%) | Included as Primary Criterion |
|---|---|---|
| Annual MS patient volume | 96.5% | ✓ |
| Number of neurologists | 93.9% | ✓ |
| Number of nurses | 91.4% | ✓ |
| Presence of physiotherapists | 90.4% | ✓ |
| Access to MRI | 98.0% | ✓ |
| Access to immunology services | 97.5% | ✓ |
| Access to neuropsychology | 90.9% | ✓ |
| Access to rehabilitation services | 91.4% | ✓ |
| Continuing medical education (CME) | 92.4% | ✓ |
| Participation in research | 90.9% | ✓ |
| Day hospital availability | 95.5% | ✓ |
| Access to clinical psychology | 94.4% | ✓ |
| Outpatient consultation area | 97.5% | ✓ |
| EDSS registry | 94.9% | ✓ |
| Patient communication system | 97.5% | ✓ |
| Access to DMTs | 100.0% | ✓ |
| Area for extraction/manipulation of fluids | 90.4% | |
| Biobanking (Sera/CSF) | 91.9% | |
| Administrative data system | 92.4% | |
| Cost accountability system | 90.4% | |
| MS patient registry | 100.0% | ✓ |
| Assessment of patient satisfaction | 91.4% | ✓ |

## Table S15c. Summary of Final MSCU Classification Criteria of Hospital MS-Centers (N=168)

| Criterion | Response Rate (%) | Primary Criterion |
|---|---|---|
| MS patient volume (annual) | 96.5 | Yes |
| Number of neurologists | 93.9 | Yes |
| Number of nurses | 91.4 | Yes |
| Presence of physiotherapists | 90.4 | Yes |
| Access to MRI | 98.0 | Yes |
| Access to immunology services | 97.5 | Yes |
| Access to neuropsychology | 90.9 | Yes |
| Access to rehabilitation services | 91.4 | Yes |
| Continuing medical education (CME) | 92.4 | Yes |
| Participation in research | 90.9 | Yes |
| Day hospital availability | 95.5 | Yes |
| Access to clinical psychology | 94.4 | Yes |
| Outpatient consultation area | 97.5 | Yes |
| EDSS registry | 94.9 | Yes |
| Patient communication system | 97.5 | Yes |
| Access to DMTs | 100.0 | Yes |
| Area for extraction/manipulation of fluids | 90.4 | |
| Biobanking (Sera/CSF) | 91.9 | |
| Administrative data system | 92.4 | |
| Cost accountability system | 90.4 | |
| MS patient registry | 95.5 | Yes |
| Assessment of patient satisfaction | 91.4 | Yes |

## Table S15d. Compliance with MSCU Criteria by Hospital type and Region (N=168)

| Criterion | Region | Institution Type | MSCU-I (%) | MSCU-II (%) |
|---|---|---|---|---|
| MS patient volume (annual) | Western Europe | Academic | 84.4 | 79.0 |
| MS patient volume (annual) | Western Europe | Non-Academic | 93.3 | 72.0 |
| MS patient volume (annual) | Other European Countries | Academic | 78.9 | 63.1 |
| MS patient volume (annual) | Other European Countries | Non-Academic | 76.5 | 77.3 |
| MS patient volume (annual) | Latin America | Academic | 90.0 | 74.2 |
| MS patient volume (annual) | Latin America | Non-Academic | 75.5 | 79.4 |
| Number of neurologists | Western Europe | Academic | 95.8 | 64.2 |
| Number of neurologists | Western Europe | Non-Academic | 79.5 | 63.7 |
| Number of neurologists | Other European Countries | Academic | 82.6 | 70.5 |
| Number of neurologists | Other European Countries | Non-Academic | 85.8 | 65.8 |
| Number of neurologists | Latin America | Academic | 90.3 | 62.8 |
| Number of neurologists | Latin America | Non-Academic | 82.3 | 67.3 |
| Number of nurses | Western Europe | Academic | 86.4 | 75.7 |
| Number of nurses | Western Europe | Non-Academic | 80.0 | 70.3 |
| Number of nurses | Other European Countries | Academic | 89.8 | 60.9 |
| Number of nurses | Other European Countries | Non-Academic | 90.2 | 63.4 |
| Number of nurses | Latin America | Academic | 76.6 | 79.0 |
| Number of nurses | Latin America | Non-Academic | 99.1 | 76.2 |
| Presence of physiotherapists | Western Europe | Academic | 82.6 | 62.0 |
| Presence of physiotherapists | Western Europe | Non-Academic | 92.1 | 68.8 |
| Presence of physiotherapists | Other European Countries | Academic | 78.1 | 69.9 |
| Presence of physiotherapists | Other European Countries | Non-Academic | 75.9 | 78.2 |
| Presence of physiotherapists | Latin America | Academic | 81.5 | 73.3 |
| Presence of physiotherapists | Latin America | Non-Academic | 82.8 | 70.4 |
| Access to MRI | Western Europe | Academic | 88.7 | 63.7 |
| Access to MRI | Western Europe | Non-Academic | 99.2 | 75.5 |
| Access to MRI | Other European Countries | Academic | 98.5 | 77.9 |
| Access to MRI | Other European Countries | Non-Academic | 89.9 | 78.4 |
| Access to MRI | Latin America | Academic | 77.2 | 63.9 |
| Access to MRI | Latin America | Non-Academic | 76.1 | 66.5 |

## Table S15e. Summary Table of Criteria for MSCU-I / MSCU-II Classification with Minimum Thresholds

| Criterion | Variable Type | Essential for MSCU-I | Indicative Threshold | Essential for MSCU-II | Indicative Threshold |
|---|---|---|---|---|---|
| Annual volume of MS patients | Continuous quantitative | Yes | ≥1500 | Yes | ≥1000 |
| Number of neurologists | Discrete quantitative | Yes | ≥5 | Yes | ≥3 |
| Number of nurses | Discrete quantitative | Yes | ≥4 | No | - |
| Presence of physiotherapists | Binary (Yes/No) | Yes | - | Yes | - |
| Access to MRI | Binary | Yes | - | Yes | - |
| Access to immunology services | Binary | Yes | - | Yes | - |
| Access to neuropsychology | Binary | No | - | Yes | - |
| Access to rehabilitation services | Binary | Yes | - | Yes | - |
| Continuing medical education (CME) | Binary | Yes | - | No | - |
| Participation in research | Binary | Yes | - | Yes | - |
| Day hospital availability | Binary | Yes | - | Yes | - |
| Access to clinical psychology | Binary | Yes | - | Yes | - |
| Outpatient consultation area | Binary | Yes | - | Yes | - |
| EDSS registry | Binary | Yes | - | Yes | - |
| Patient communication system | Binary | No | - | Yes | - |
| Access to DMTs | Binary | Yes | - | Yes | - |
| Area for fluid extraction/handling | Binary | No | - | No | - |
| Biobanking (sera/CSF) | Binary | No | - | No | - |
| Administrative data system | Binary | No | - | No | - |
| Cost accountability system | Binary | No | - | No | - |
| MS patient registry | Binary | Yes | - | Yes | - |
| Assessment of patient satisfaction | Binary | Yes | - | Yes | - |

Note: This scoring model differs from the original classification (120 MSCU-I, 21 MSCU-II) based on proportional fulfillment of 22 criteria, as described in the main Results section.

## Table S15f. Automatic Scoring Scheme for MSCU Classification

| Criterion | Variable Type | MSCU-I Requirement | MSCU-II Requirement |
|---|---|---|---|
| Total essential criteria | Score threshold | ≥14 out of 16 | ≥14 out of 16 |
| Annual MS patient volume | Continuous quantitative | ≥1500 | ≥1000 |
| Number of neurologists | Discrete quantitative | ≥5 | ≥3 |
| Number of nurses | Discrete quantitative | ≥4 | Not required |
| Presence of physiotherapist | Binary (Yes/No) | Yes | Yes |
| Access to MRI | Binary (Yes/No) | Yes | Yes |
| Access to immunology services | Binary (Yes/No) | Yes | Yes |
| Access to neuropsychology | Binary (Yes/No) | Not required | Yes |
| Access to rehabilitation services | Binary (Yes/No) | Yes | Yes |
| Continuing medical education (CME) | Binary (Yes/No) | Yes | Not required |
| Participation in research | Binary (Yes/No) | Yes | Yes |
| Day hospital availability | Binary (Yes/No) | Yes | Yes |
| Access to clinical psychology | Binary (Yes/No) | Yes | Yes |
| Outpatient consultation area | Binary (Yes/No) | Yes | Yes |
| EDSS registry | Binary (Yes/No) | Yes | Yes |
| Patient communication system | Binary (Yes/No) | Not required | Yes |
| Access to DMTs | Binary (Yes/No) | Yes | Yes |
| MS patient registry | Binary (Yes/No) | Yes | Yes |
| Assessment of patient satisfaction | Binary (Yes/No) | Yes | Yes |

Note: One point is assigned for each essential criterion fulfilled. This scoring system enables an automated and objective classification of MS Care Units (MSCUs) based on the availability of essential clinical, human, and structural resources. This scoring model differs from the original classification (120 MSCU-I, 21 MSCU-II), which was based on proportional fulfilment of 22 predefined criteria (≥80% for MSCU-I; 70–79% for MSCU-II), as detailed in the main Results section.

## Table S16. Assessment of User Satisfaction by Region and Type of Hospital (N=168)

| Region | Academic Hospital (%) | Non-Academic Hospital (%) |
|---|---|---|
| Western European Countries | 81.3 | 75.7 |
| Other European Countries | 76.9 | 73.3 |
| North America | 50.0 | 75.0 |
| Latin America | 46.2 | 55.6 |
| Rest of the World | 33.3 | 50.0 |

## Table S17. Distribution of Patient-Reported Outcome Assessments by Region and Hospital Type (absolute number) (N=168)

Data represents the number of MS-Centers reporting the use of each type of Patient-Reported Outcome (PRO) assessment tool. Values are counts, not percentages.

| PRO Assessment (Count) | Western Europe - Academic | Western Europe - Non-Academic | Other Europe - Academic | Other Europe - Non-Academic | North America - Academic | North America - Non-Academic | Latin America - Academic | Latin America - Non-Academic | Rest of World - Academic | Rest of World - Non-Academic |
|---|---|---|---|---|---|---|---|---|---|---|
| QoL Assessment (Count) | 38 | 34 | 21 | 18 | 6 | 5 | 10 | 14 | 3 | 2 |
| Fatigue Assessment (Count) | 29 | 30 | 17 | 12 | 4 | 4 | 6 | 8 | 2 | 1 |
| Anxiety/Depression (Count) | 17 | 12 | 14 | 11 | 2 | 2 | 3 | 4 | 1 | 1 |
| Other (Count) | 14 | 9 | 8 | 6 | 1 | 1 | 2 | 3 | 1 | 1 |
| TOTAL (Count) | 98 | 85 | 60 | 47 | 13 | 12 | 21 | 29 | 7 | 5 |

## Table S18. Distribution of Patient-Reported Outcome Assessments by Region and Hospital Type (%) (N=168)

| PRO Assessment (%) | Western Europe - Academic | Western Europe - Non-Academic | Other Europe - Academic | Other Europe - Non-Academic | North America - Academic | North America - Non-Academic | Latin America - Academic | Latin America - Non-Academic | Rest of World - Academic | Rest of World - Non-Academic |
|---|---|---|---|---|---|---|---|---|---|---|
| QoL Assessment (%) | 38.8 | 40.0 | 35.0 | 38.3 | 46.2 | 41.7 | 47.6 | 48.3 | 42.9 | 40.0 |
| Fatigue Assessment (%) | 29.6 | 35.3 | 28.3 | 25.5 | 30.8 | 33.3 | 28.6 | 27.6 | 28.6 | 20.0 |
| Anxiety/Depression (%) | 17.3 | 14.1 | 23.3 | 23.4 | 15.4 | 16.7 | 14.3 | 13.8 | 14.3 | 20.0 |
| Other (%) | 14.3 | 10.6 | 13.3 | 12.8 | 7.7 | 8.3 | 9.5 | 10.3 | 14.3 | 20.0 |
| TOTAL (%) (%) | 100.0 | 100.0 | 100.0 | 100.0 | 100.0 | 100.0 | 100.0 | 100.0 | 100.0 | 100.0 |

## Table S19. GDP and Health Expenditure per Capita by MSCU Type

| Variable | MSCU-I (n=120) | MSCU-II (n=21) |
|---|---|---|
| GDP per capita (USD) | 32,466 ± 14,467 | 12,802 ± 7,871 |
| Health Exp per capita (USD) | 3,639 ± 1,746 | 1,568 ± 925 |

## Supplementary Figures


- Figure S1. Proportional distribution of hospitals and universities across the surveyed geographic regions (N=198).
- Figure S2. Distribution of MS-Center Staff by Region and Type of Organization
- Figure S3. Heatmap illustrating the utilization of diagnostic tests by type of hospital and region (N=168).
- Figure S4. Use of Immunomodulatory Treatments by Region (N=168).
- Figure S5. Use of Immunosuppressant Treatments by Region and and Hospital Type (N=168).
- Figure S6. Distribution of MS Therapy Use by Efficacy Level, Hospital Type, and Region
- Figure S7. Availability of External Resources by Region and Hospital Type (Top 9) (N=168).
- Figure S8. Use of Communication Systems with Patients by Hospital Type (Ordered by Total Usage) (N=168).
- Figure S9. Assessment of User Satisfaction by Region and Hospital Type (N=168).
- Figure S10. Distribution of Patient-Reported Outcome Assessments by Region and Hospital Type (N=168).
- Figure S11. GDP per capita by MSCU Type
- Figure S12. Health Expenditure per capita by MSCU Type

**Figure S1. Proportional distribution of hospitals and universities across the surveyed geographic regions (N=198).**

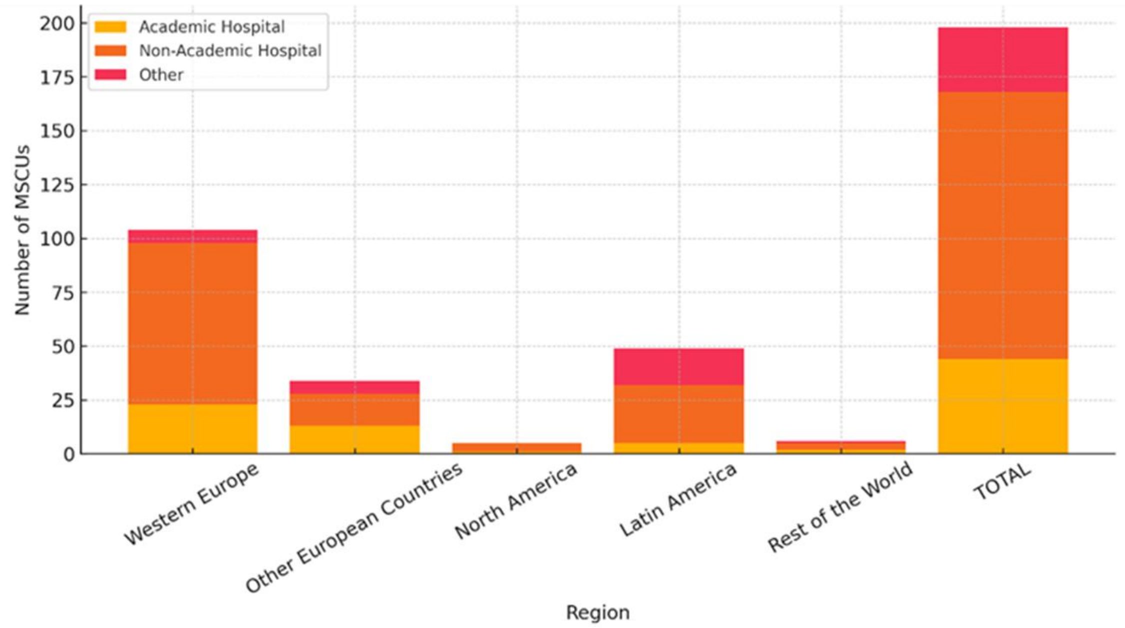

## Figure S2. Distribution of MS-Center Staff by Region and Type of Hospital (N=168)

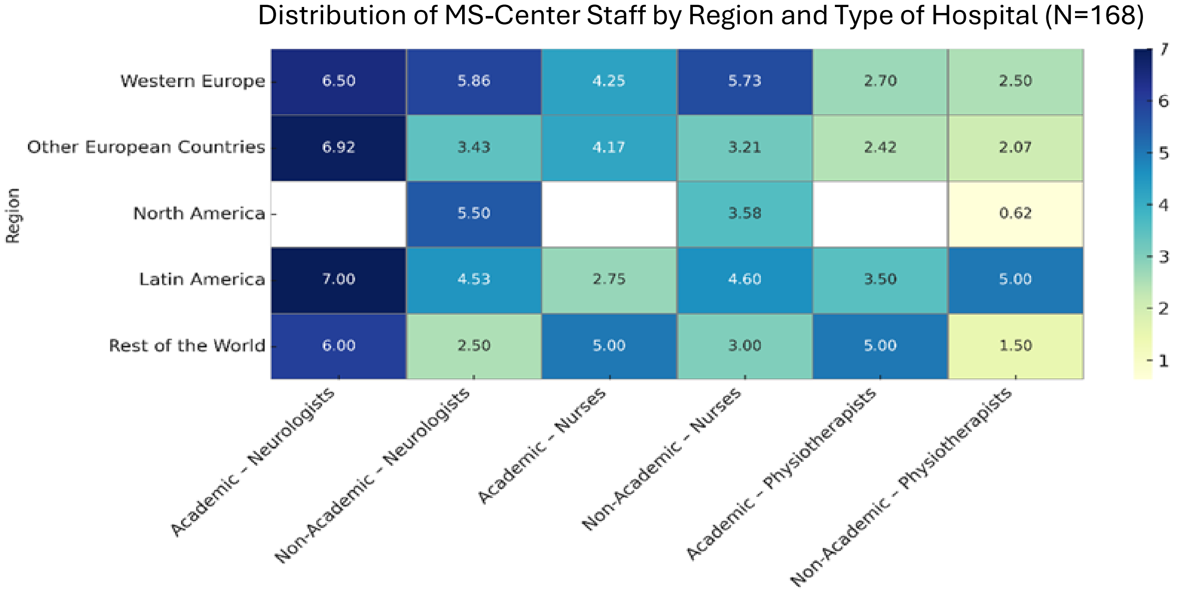

## Figure S3. Heatmap illustrating the utilization of diagnostic tests by type of hospital and region (N=168).

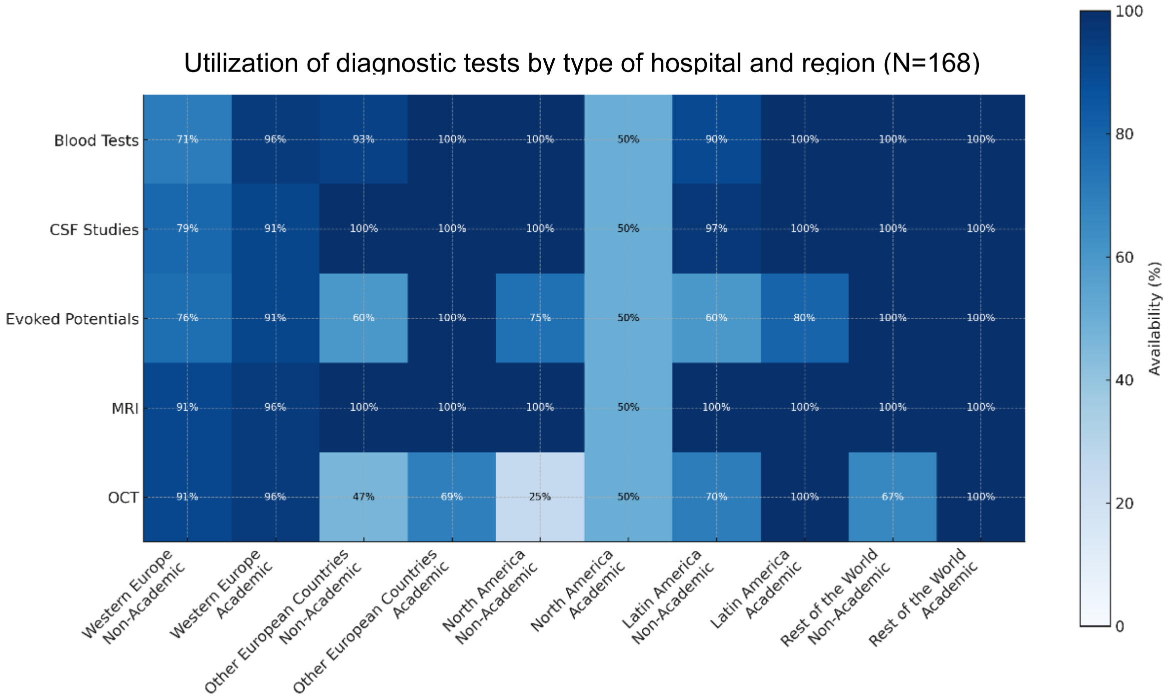


Note: Heatmap showing the availability of key diagnostic tools used in the evaluation of patients with multiple sclerosis, stratified by hospital type (Academic vs Non-Academic) and region. Availability is expressed as the percentage of MS Care Units reporting routine use of each modality: MRI, cerebrospinal fluid (CSF) analysis, evoked potentials (EPs), optical coherence tomography (OCT), and blood tests. Dark blue indicates high availability, while lighter shades indicate more limited use. MRI and blood tests were generally widespread, although not universal, whereas the use of EPs and OCT varied more significantly across regions and organization types.

## Figure S4. Use of Immunomodulatory Treatments by Region (N=168)

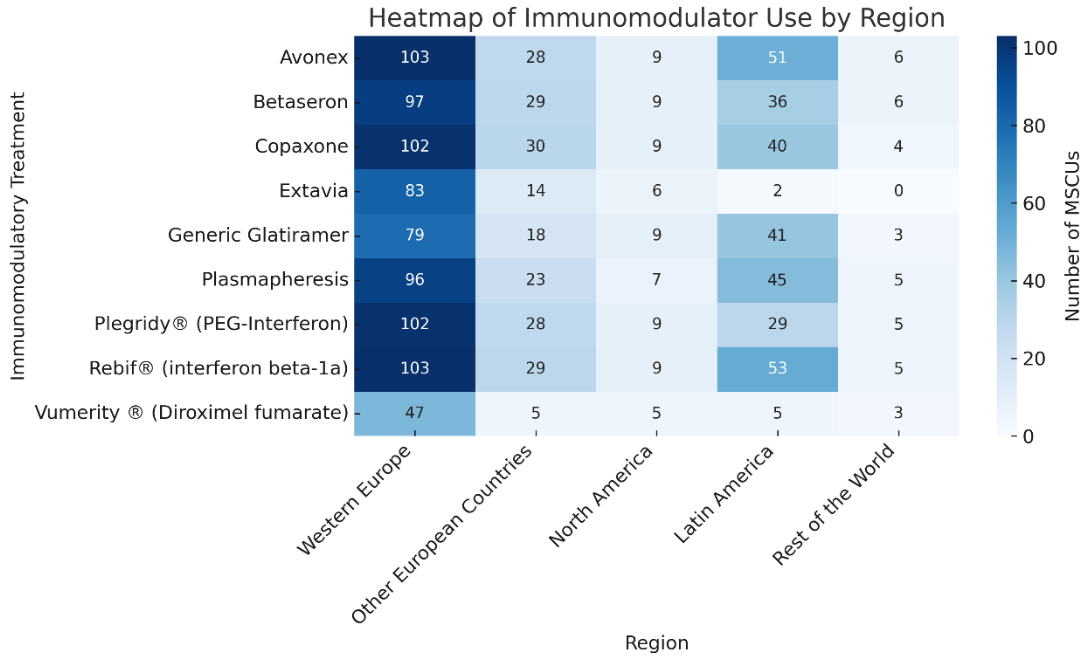


Note: Heatmap showing the number of MS-Centers Care Units (MSCUs) reporting the use of key immunomodulatory treatments across regions. Darker shades indicate more frequent usage. Data represent counts of units reporting each agent.
No statistically significant differences were observed in treatment use between Academic and Non-Academic Hospitals ($p$ > 0.14 for all major agents).

## Figure S5. Use of Immunosuppressant Treatments by Region and Hospital Type (N=168)

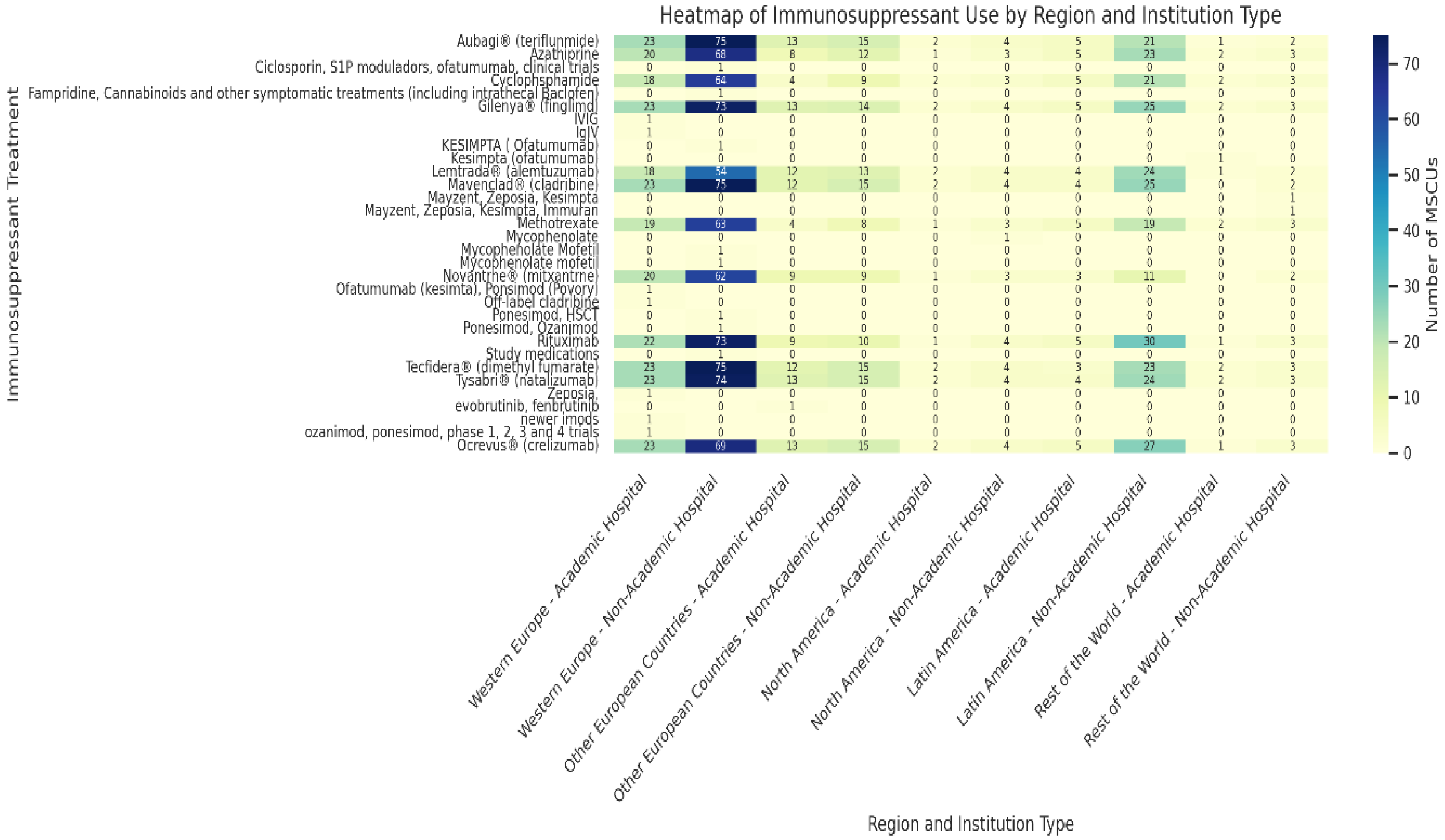

Note: Heatmap showing the number of MS-Centers reporting the use of each immunosuppressant therapy, stratified by region and organization type (Academic vs Non-Academic Hospitals).Darker colors represent higher reporting frequencies.No statistically significant differences in treatment use were observed between academic and non-academic settings (final column in table shows *p*-values from chi-square tests).Frequent therapies included **Aubagio (teriflunomide)**, **azathioprine**, and **cyclophosphamide**, with wide usage across multiple regions.

## Figure S6. Distribution of MS Therapy Use by Efficacy Level, Hospital Type, and Region

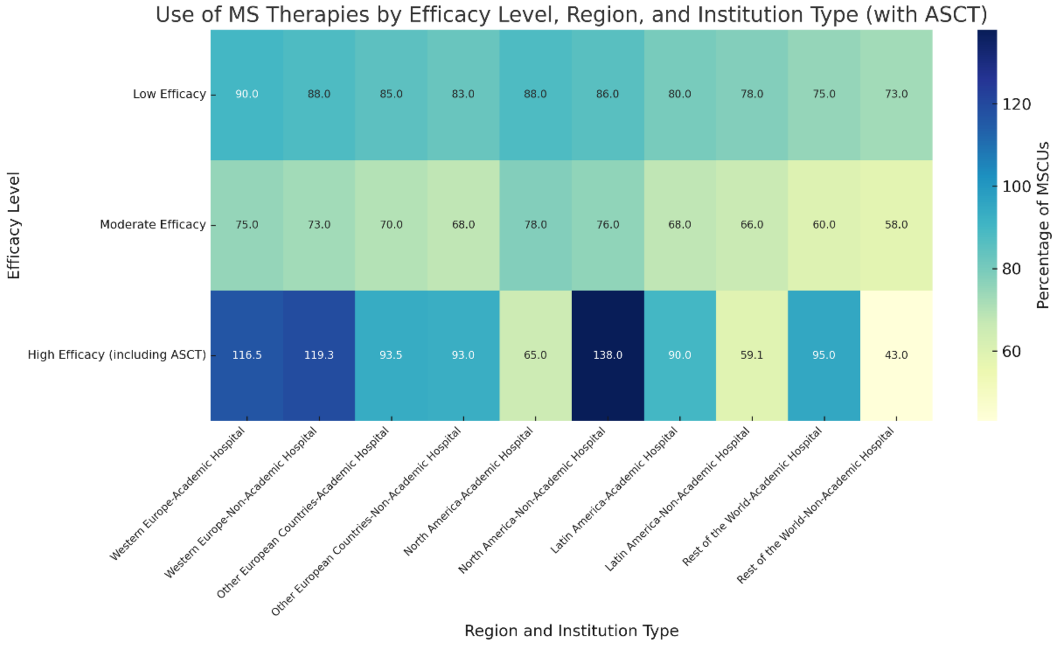


Note: Percentages by efficacy level are not mutually exclusive. A single MS-Center may report the use of therapies from multiple efficacy categories (e.g., both moderate- and high-efficacy DMTs), and therefore, totals across efficacy levels may exceed 100%.

## Figure S7. Availability of External Resources by Region and Hospital Type (Top 9) (N=168)

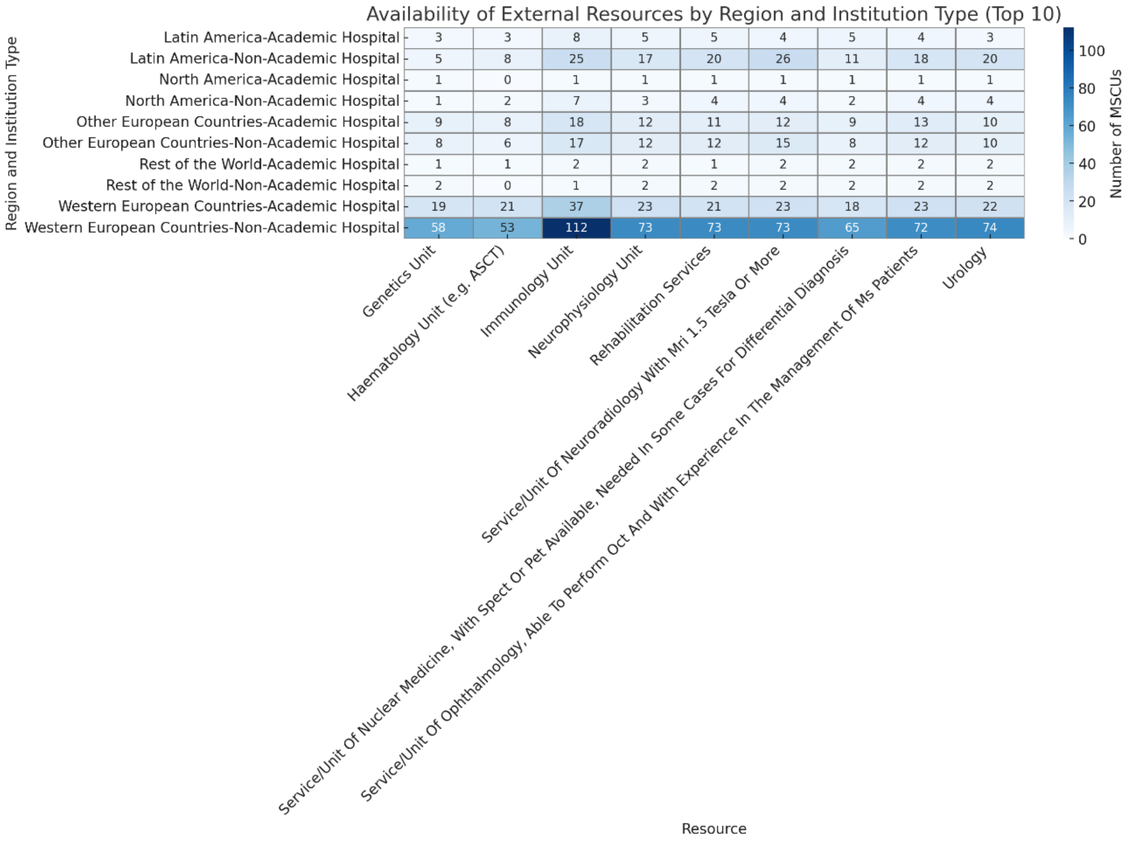

## Figure S8. Use of Communication Systems with Patients by Hospital Type (Ordered by Total Usage)(N=168)

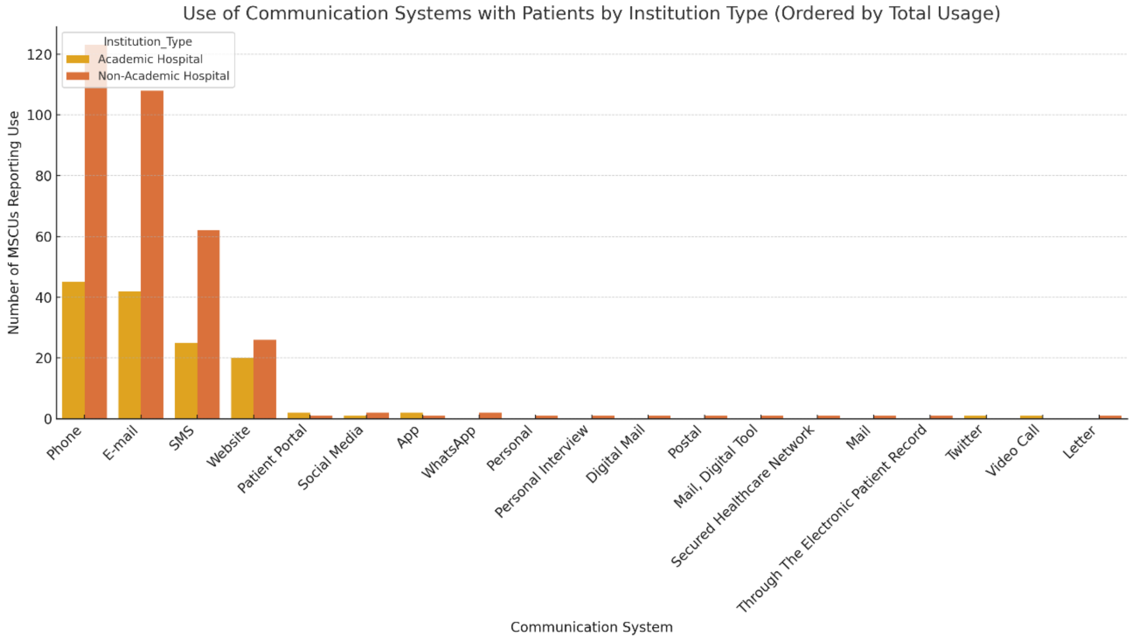

## Figure S9. Assessment of User Satisfaction by Region and Hospital Type (N=168)

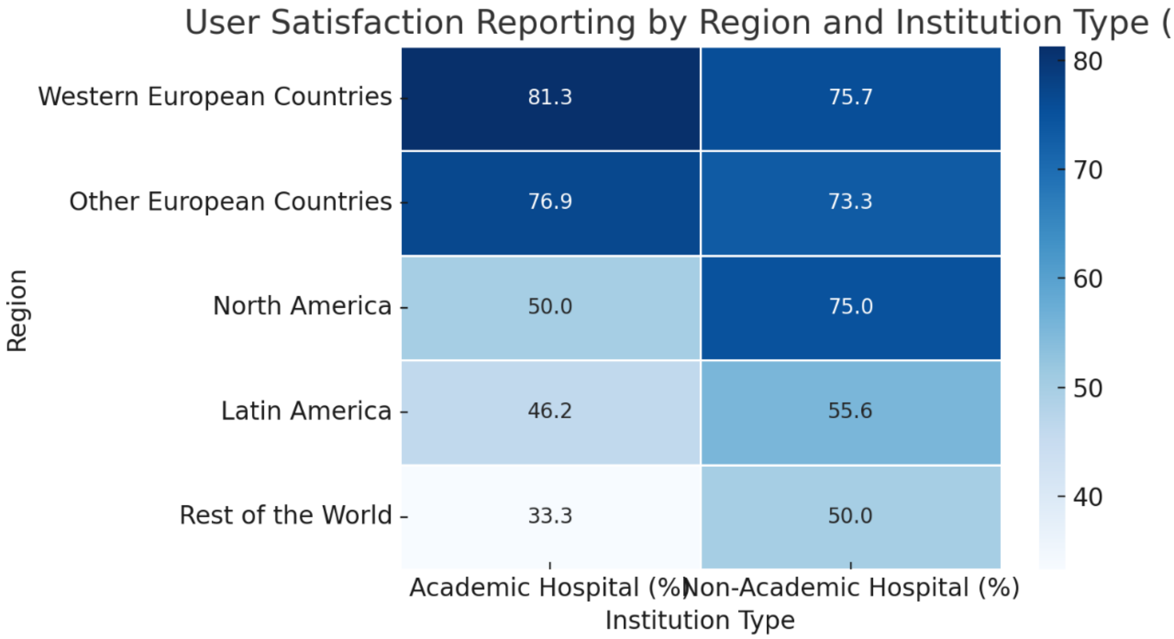


Heatmap showing the percentage of MS-Centers reporting routine assessment of user satisfaction, stratified by global region and type of organization (Academic vs Non-Academic Hospitals). Darker blue tones represent higher reporting rates. No statistically significant differences were found across settings (p = 0.856, chi-square test).

## Figure S10. Distribution of Patient-Reported Outcome Assessments by Region and Hospital Type (N=168)

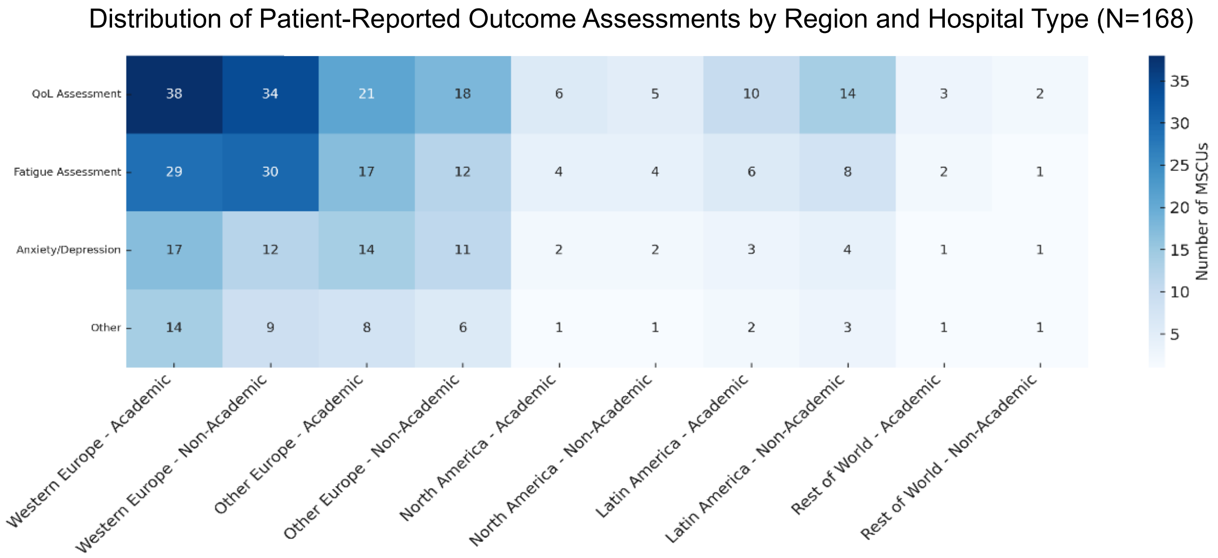

## Figure S11. GDP per capita by MSCU Type

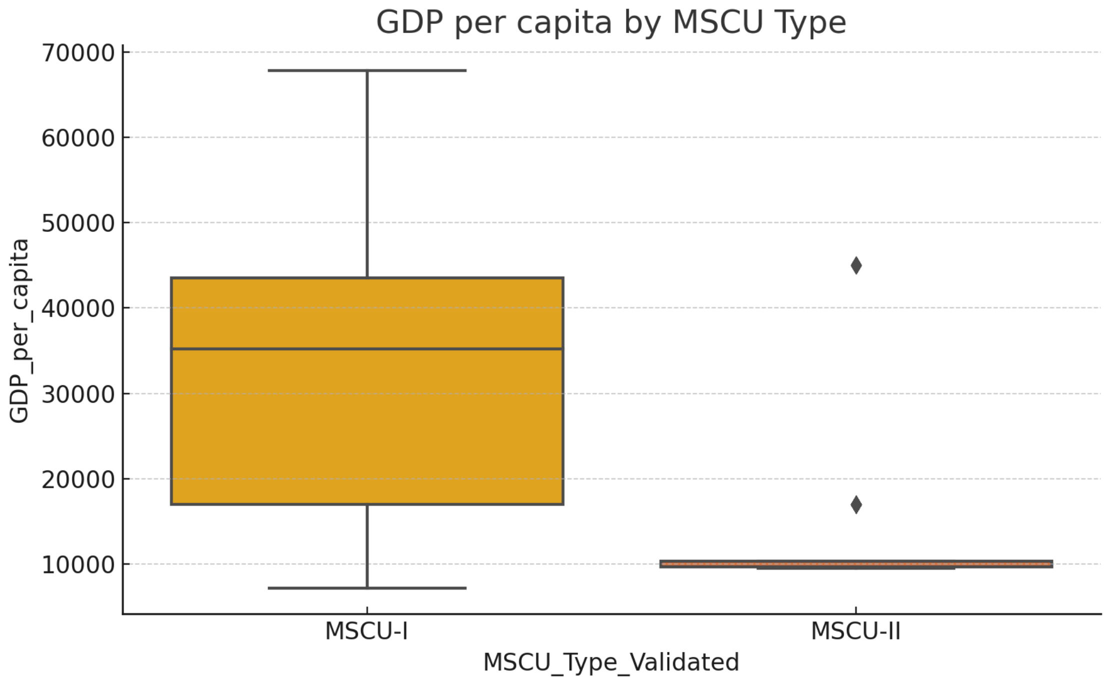

## Figure S12. Health Expenditure per capita by MSCU Type

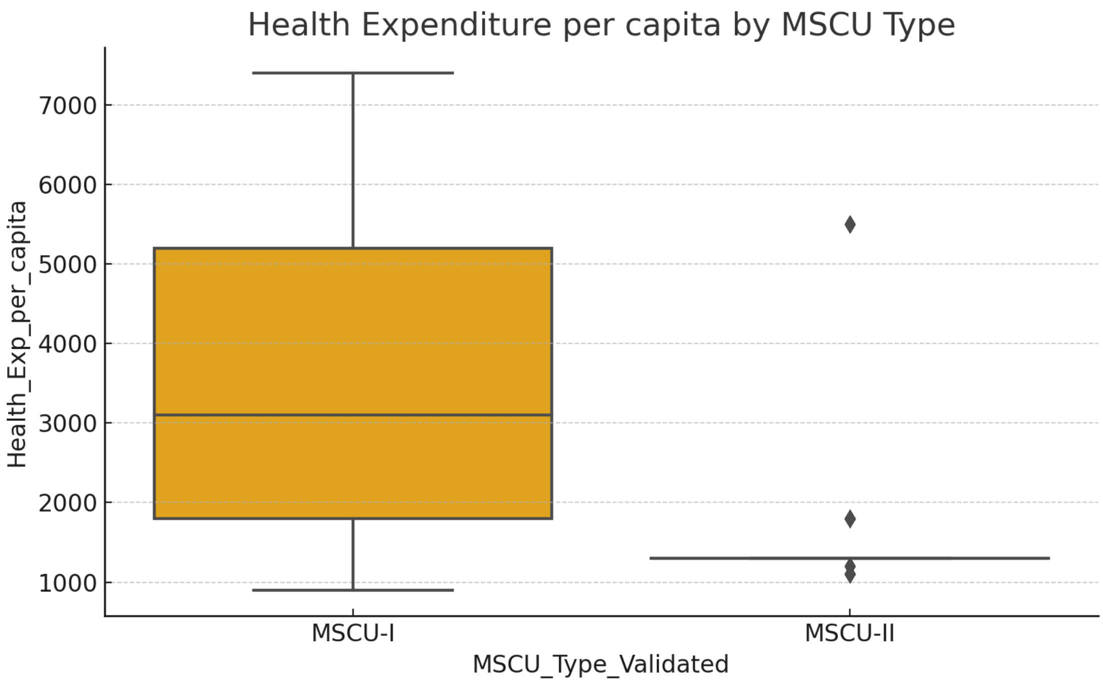

Supplement: Supplementary file 1 [file Data_Sheet_1.pdf]
